# Dual-emissive 2-(2′-hydroxyphenyl)oxazoles for high performance organic electroluminescent devices: discovery of a new equilibrium of excited state intramolecular proton transfer with a reverse intersystem crossing process†
†Electronic supplementary information (ESI) available: Synthetic procedures, spectroscopic data, physicochemical properties, device performances, and computational details. See DOI: 10.1039/c7sc04464j


**DOI:** 10.1039/c7sc04464j

**Published:** 2017-12-01

**Authors:** Bijin Li, Linsen Zhou, Hu Cheng, Quan Huang, Jingbo Lan, Liang Zhou, Jingsong You

**Affiliations:** a Key Laboratory of Green Chemistry and Technology of Ministry of Education , College of Chemistry , Sichuan University , 29 Wangjiang Road , Chengdu 610064 , PR China . Email: jsyou@scu.edu.cn ; Email: jingbolan@scu.edu.cn; b State Key Laboratory of Rare Earth Resource Utilization , Changchun Institute of Applied Chemistry , Chinese Academy of Sciences , 5625 Renmin Street , Changchun 130022 , PR China . Email: zhoul@ciac.ac.cn; c Center of Interface Dynamics for Sustainability , Institute of Materials , China Academy of Engineering Physics , 596 Yinhe Road , Chengdu 610200 , PR China

## Abstract

The photoluminescence (PL) and electroluminescence (EL) properties of two highly efficient excited state intramolecular proton transfer (ESIPT) molecules, 2-(2′-hydroxyphenyl)oxazoles containing one triphenylamine (TPA) (**1**) and two TPAs (**2**) respectively, are studied systematically. The enol-forms of both **1** and **2** possess highly hybridized local and charge transfer (HLCT) excited state character, while their excited-state keto-forms are not of obvious HLCT character. A **1**-based device exhibits green-white electroluminescence with Commission Internationale d’Eclairage (CIE) coordinates of (0.25, 0.41) and a high external quantum efficiency (EQE) up to 5.3%, which is the highest EQE value recorded for single molecular white light-emitting materials. A **2**-based device shows sky-blue emission with CIE coordinates of (0.18, 0.16) and an EQE of 8.0%, which is the highest EQE in the reported HLCT materials. The fluorescence intensities of the enol-forms of **1** and **2** in EL spectra are increased remarkably relative to their PL spectra. Experimental data and theoretical calculations reveal a new ESIPT equilibrium with a reverse intersystem crossing (RISC) process arising from the HLCT character. In EL, the RISC of the enol-form excitons from the triplet state to the singlet state triggers an increase in the number of enol-form singlet excitons, which further leads to a shift of the ESIPT equilibrium towards an enhanced enol-form emission. Thus, the difference between the ESIPT equilibria in PL and EL may be ascribed to the HLCT character of the enol-form excited state.

## Abstract 2

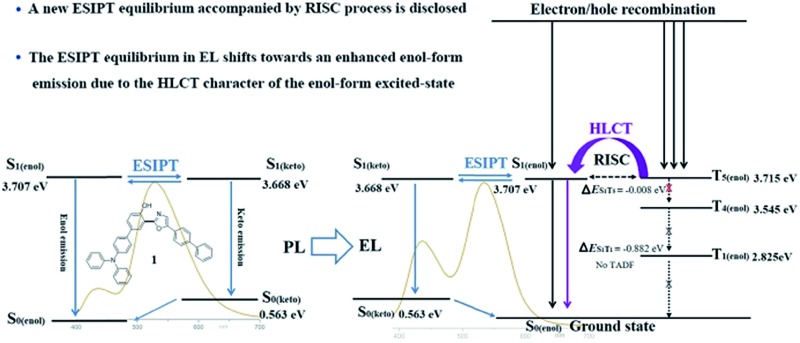
Dual-emission of the enol-forms and keto-forms of 2-(2′-hydroxyphenyl)oxazoles and their application in OLEDs are investigated.

## Introduction

White organic light-emitting devices (WOLEDs) have been attracting much research interest due to their widespread use in flat panel displays and illuminations.^1^,^2^ The most common method to attain white luminescence is to combine two or three emitting colors (blue/orange or blue/green/red) from different emitting centers.^1^ However, mixing two or three emissive materials usually requires the maintenance of a delicate balance between chromophores, in terms of excitation and emission, to fabricate a white light-emitting device.2*c* Moreover, a dual or multi-layer system from multiple molecules with different emission profiles would accentuate the difficulty in device fabrication.^1^ In addition, the differential photostability of multiple emitters may give rise to the variation of emitting colors in working devices upon long-term use, which makes the chromaticity control very challenging.1e,2c Compared to combined emitters, single-molecular white-light-emitting materials are likely to offer several advantages, such as long-term color balance, stability and simple fabrication processes.^2^

Excited state intramolecular proton transfer (ESIPT) involves an electric field or photo-induced keto–enol tautomerization process, which offers an opportunity for the dual-emission of the enol-form and keto-form, and thus organic molecules with ESIPT characteristics are regarded as ideal candidates for single molecular white light-emitting materials.2d,g,^3^,^4^ However, the establishment of the ESIPT equilibrium between the enol-form and keto-form emissions remains highly challenging. Moreover, the excited state equilibrium in electroluminescence (EL) is usually different from that in photoluminescence (PL), and the mechanism causing this phenomenon is still unclear, which further increases the difficulty to access high performance white-light devices.2d,g,^3^ In addition, ESIPT molecules usually exhibit relatively low photoluminescence (PL) efficiency and external quantum efficiency (EQE) in electroluminescent (EL) devices due to the unfavorable non-radiative decay route as well as the theoretical upper limit of the singlet exciton ratio of 25% for conventional fluorescent materials.2d,g,^3^

The hybridized local and charge transfer (HLCT) excited state is characterized as the coexistence or hybridization of locally excited (LE) and charge transfer (CT) states, and is one of three main models that can break through the singlet exciton statistics limit.^5^ The LE state can usually provide a high PL efficiency due to an almost complete HOMO/LUMO overlap and a consequently large transition moment.^5^ The CT state facilitates a small singlet-triplet energy level splitting (Δ*E*_ST_), which offers an opportunity for high singlet exciton yields through reverse intersystem crossing (RISC) from the triplet state to the singlet state.^5^ Recently, many organic EL materials with the HLCT excited state character have been developed with excellent EQEs and high singlet exciton yields through the efficient RISC.^5^ However, organic light-emitting materials with both the HLCT excited state character and the dual-emission behaviour arising from ESIPT have not yet been described. In this work, we systematically investigate the PL and EL properties of two highly efficient ESIPT molecules, 2-(2′-hydroxyphenyl)oxazoles containing one triphenylamine (TPA) (**1**) and two TPAs (**2**) respectively, and disclose a new ESIPT equilibrium accompanied by an RISC process.

## Results and discussions

### Synthesis

**Scheme 1:**
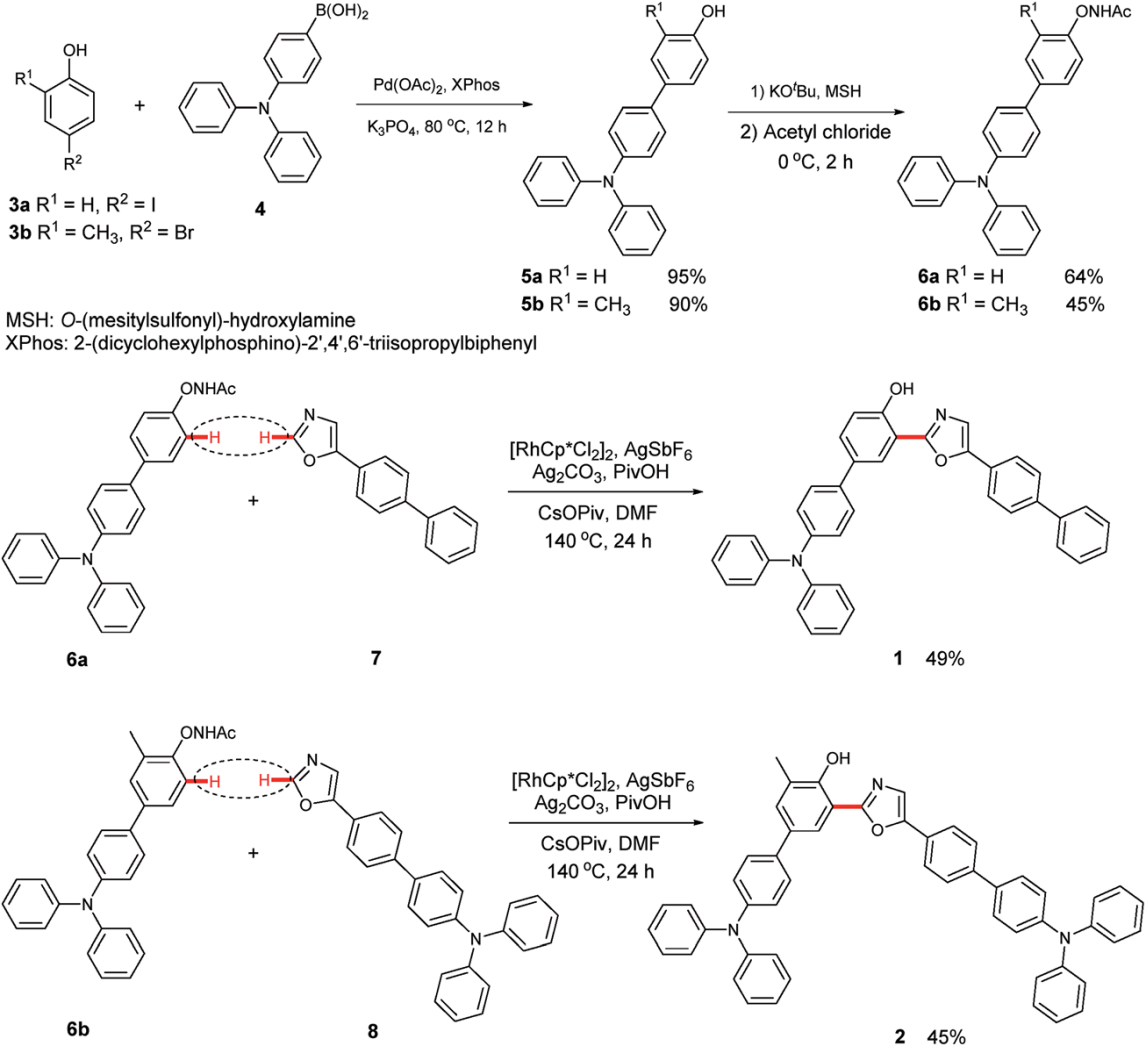
Synthetic routes of **1** and **2**.

The synthetic route to 3-(5-([1,1′-biphenyl]-4-yl)oxazol-2-yl)-4′-(*N*,*N*-diphenylamino)-[1,1′-biphenyl]-4-ol (**1**) is shown in Scheme 1. First, 4′-(*N*,*N*-diphenylamino)-[1,1′-biphenyl]-4-ol (**5a**) was obtained in a good yield by the Suzuki coupling of 4-iodophenol (**3a**) with 4-(*N*,*N*-diphenylamino)-1-phenylboronic acid (**4**).4*b* Next, *N*-((4′-(*N*,*N*-diphenylamino)-[1,1′-biphenyl]-4-yl)oxy)acetamide (**6a**) was prepared *via* a transamination reaction with *O*-(mesitylsulfonyl)hydroxylamine (MSH) as the aminating reagent and an *N*-acetylation with acyl chloride.^6^ Finally, the target product (**1**) was synthesized by the Rh(iii)-catalyzed oxidative C–H/C–H cross-coupling reaction of **6a** with 5-([1,1′-biphenyl]-4-yl)oxazole (**7**). Utilizing a similar synthetic strategy, 4′-(*N*,*N*-diphenylamino)-3-(5-(4′-(diphenylamino)-[1,1′-biphenyl]-4-yl)oxazol-2-yl)-5-methyl-[1,1′-biphenyl]-4-ol (**2**) was prepared (Section III, ESI†).

### Photophysical properties

The absorption spectra of compounds **1** and **2** were measured in toluene (Fig. S1†), and their absorption maxima are summarized in Table 1. Their absorption bands in the short-wavelength region correspond to the π–π* transition or the n–π* transition, while the longest wavelength absorption can be assigned to an intramolecular charge transfer (ICT) transition.5g,^7^ The longest wavelength absorption maximum of **2** at 378 nm is red-shifted by 9 nm relative to the absorption shoulder of **1** at 369 nm and the absorption intensity is increased significantly (*ε*_(_**_1_**_, 369 nm)_ = 1.5 × 10^4^ M^–1^ cm^–1^; *ε*_(_**_2_**_, 378 nm)_ = 3.6 × 10^4^ M^–1^ cm^–1^), indicating a relatively stronger ICT character for **2**.7b,c

**Table 1:** The thermal and electrochemistry data of **1** and **2**^*a*^

Compounds	*λ*_abs_ (nm)	*λ*_enol-em_ (nm)	*λ*_keto-em_ (nm)	CIE_1931_	HOMO^*b*^ (eV)	LUMO^*b*^ (eV)	*T*_d_ ^*c*^ (°C)
**1**	321, 335, 369	434	532	(0.32, 0.45)	–5.27	–2.10	381
**2**	310, 335, 378	424	535	(0.27, 0.33)	–5.22	–2.27	430

^*a*^Absorption and emission maxima were measured in toluene (5.0 × 10^–5^ M). *λ*_enol-em_ = enol-form emission maximum, and *λ*_keto-em_ = keto-form emission maximum.

^*b*^The estimated value based on cyclic-voltammetry data and optical bandgaps.

^*c*^The temperature for 5% weight loss of materials.

**Fig. 1:**
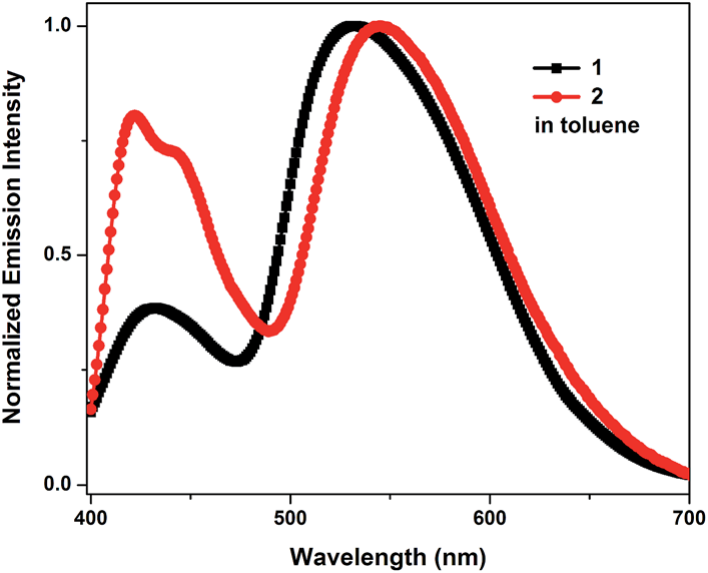
Normalized emission spectra of **1** and **2** in toluene (5.0 × 10^–5^ M).

The emission spectra of **1** and **2** recorded in toluene exhibit an apparent dual-emission behavior (Fig. 1). The normal enol-form emission is located in the blue region and the proton-transfer keto-form emission is positioned at the red-shifted part of the spectrum. The enol-form emission of **1** is substantially weaker than its keto-form emission. Therefore, **1** exhibits a yellowish-green emission in toluene with CIE coordinates of (0.32, 0.45) (Table 1). In contrast, **2** shows a well-balanced dual-emission between the enol-form and keto-form, which covers the whole visible range extending from 400 to 700 nm, thus leading to a nearly pure white-light emission with CIE coordinates of (0.27, 0.33).

### Electrochemical properties

The electrochemical investigation was performed *via* cyclic voltammetric (CV) experiments using an Ag/Ag^+^ reference electrode, a platinum wire counter electrode, and a platinum plate working electrode. The HOMO energy level of **1** was calculated to be –5.27 eV by comparison with the standard ferrocene/ferrocenium (Fc/Fc^+^) redox couple. The optical bandgap is deduced from its UV-vis spectrum in acetonitrile solution. The LUMO level of **1** is calculated to be –2.10 eV by subtracting the optical bandgap from the HOMO level. The HOMO and LUMO levels of **2** are –5.22 eV and –2.27 eV, respectively (Table 1 and Section V, ESI†).

### Thermal properties

The thermal properties of **1** and **2** were gauged using thermogravimetric analysis (TGA) and differential scanning calorimetry (DSC). TGA was carried out under a nitrogen atmosphere at a heating rate of 10 °C min^–1^. As demonstrated by the TGA, **1** exhibits high thermal stability with a 5% weight loss temperature up to 381 °C (Table 1 and Fig. S3†). DSC was performed in the temperature range from 50 °C to 250 °C at a heating rate of 20 °C min^–1^. DSC analysis indicates that **1** melted at 225 °C and there is no phase transformation until the sample melted completely. Compound **2**, with a higher molecular weight, showed a higher decomposition temperature of 430 °C. However, **2** possessed a low glass-transition temperature (*T*_g_) of 143 °C due to its amorphous character (Table S1, Fig. S2 and S3†).3*b*

### Electroluminescence performances

**Fig. 2:**
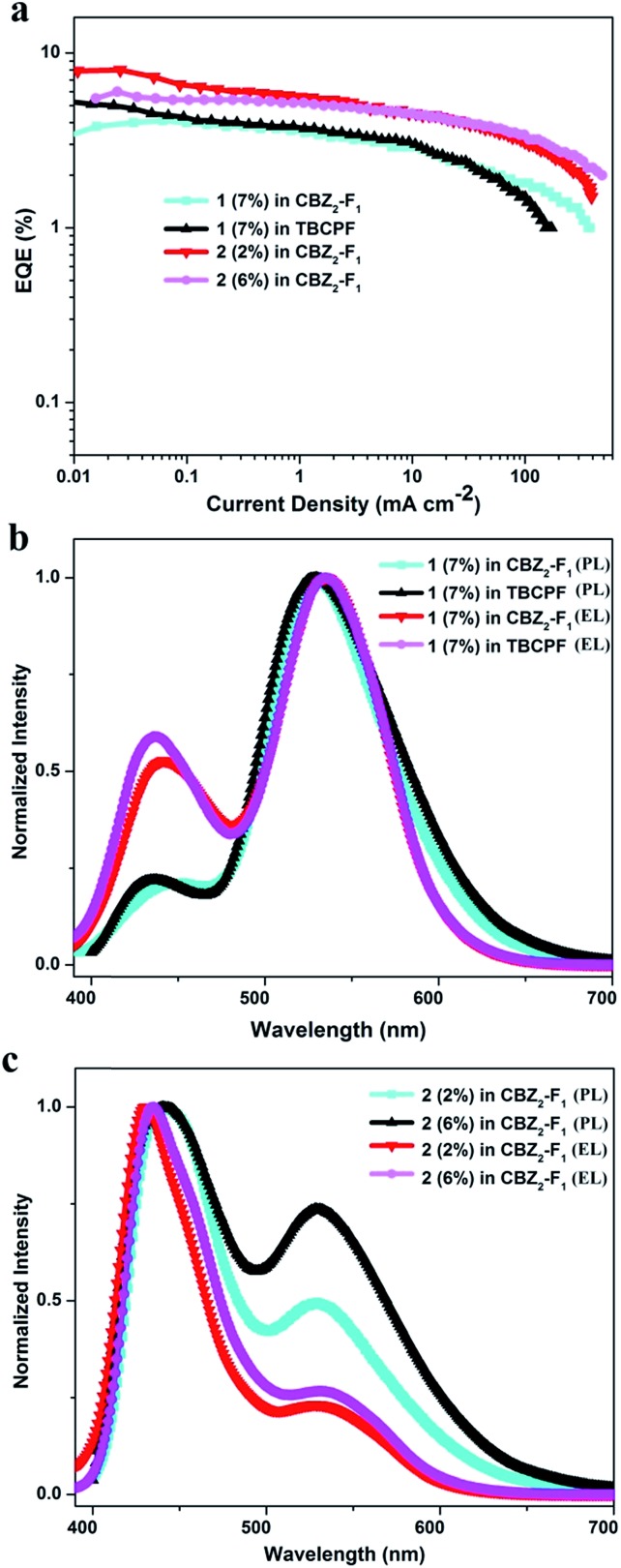
(a) EQE–current density curves of the devices based on **1** and **2**. (b) Normalized EL spectra of the **1**-based devices operating at 1 mA cm^–2^ and PL spectra of **1** as a film. (c) Normalized EL spectra of **2**-based devices operating at 1 mA cm^–2^ and PL spectra of **2** as a film.

To explore the dual-emission property of these two molecules in EL devices, devices with **1** and **2** as the emitters were fabricated. The device architectures are displayed in Fig. S6†. Indium tin oxide (ITO) acts as the anode; molybdenum oxide (MoO_3_) serves as the hole-injecting layer; 1,1-bis{(di-4-tolylamino)phenyl}cyclohexane (TAPC) acts as the hole-transporting layer; 3,3′-(9*H*-fluorene-9,9-diyl)bis(9-phenyl-9*H*-carbazole) (CBZ_2_-F_1_) is used as the host material; 1,3,5-tri(*m*-(pyrid-3-ylphenyl)benzene) (TmPyPB) or 1,3,5-tris(6-(3-(pyridin-3-yl)phenyl)pyridin-2-yl)benzene (Tm3PyP26PyB) work as the electron-transporting layer; and LiF/Al is employed as the cathode (Section VI, ESI†). Initially, devices based on different doping concentrations of **1** in CBZ_2_-F_1_ were fabricated with the device structure: ITO/MoO_3_ (3 nm)/TAPC (50 nm)/**1** (*x* wt%): CBZ_2_-F_1_ (20 nm)/TmPyPB (50 nm)/LiF (1 nm)/Al (100 nm). The device based on **1** (7 wt%) in CBZ_2_-F_1_ exhibits high performance with the maximum EQE (EQE_max_), brightness (*L*_max_), current efficiency (CE_max_), power efficiency (PE_max_) and CIE coordinates of 4.1%, 10 876 cd m^–2^, 11.77 cd A^–1^, 10.38 lm W^–1^ and (0.25, 0.42), respectively (Fig. 2a, Table 2 and S2†). Remarkably, when the host material, CBZ_2_-F_1_, is replaced with 9,9-di(4,4′-bis(3,6-di-*tert*-butyl-9*H*-carbazole)-phenyl)-9*H*-fluorene (TBCPF), the device performance is further improved. The EL device based on **1** (7 wt%) in TBCPF shows a maximum EQE up to 5.3% with CIE coordinates of (0.25, 0.41), which is the highest EQE value recorded for single molecular white light-emitting materials as emitters.^2^ The devices based on **2** (*x*%) in CBZ_2_-F_1_ were also fabricated with the structure: ITO/MoO_3_ (3 nm)/TAPC (50 nm)/**2** (*x* wt%): CBZ_2_-F_1_ (20 nm)/Tm3PyP26PyB (50 nm)/LiF (1 nm)/Al (100 nm). Surprisingly, the EL devices based on **2** exhibit sky-blue emission (Tables 2 and S2†). It is worth noting that the device based on **2** (2%) in CBZ_2_-F_1_ shows a brightness of 8101 cd m^–2^ and a maximum EQE up to 8.0%, while the maximum brightness of the device based on **2** (6%) in CBZ_2_-F_1_ reaches 14 143 cd m^–2^. Utilizing TBCPF as the host material, the EL devices based on **2** (*x*%) in TBCPF still exhibit sky-blue emission, but slightly inferior electroluminescence performance compared to those in CBZ_2_-F_1_ (Table S2†).

**Table 2:** Device performance with **1** and **2** as emitters^*a*^

Device^*a*^	*V*_turn-on_ ^*a*^ [V]	CIE_1931_^*b*^ [*x*, *y*]	EQE_max_^*c*^ [%]	CE_max_^*d*^ [cd A^–1^]	PE_max_^*e*^ [lm W^–1^]	*L*_max_ ^*f*^ [cd m^–2^]	Device performance at 1000 cd m^–2^		
							EQE [%]	CE [cd A^–1^]	PE [lm W ^–1^]
**1** (7%) in CBZ_2_-F_1_	3.4	(0.25, 0.42)	4.1	11.77	10.38	10 876	2.7	7.68	4.31
**1** (7%) in TBCPF	3.7	(0.25, 0.41)	5.3	14.54	12.34	4715	2.8	7.63	3.47
**2** (2%) in CBZ_2_-F_1_	3.5	(0.18, 0.16)	8.0	9.97	8.95	8101	4.2	5.21	2.60
**2** (6%) in CBZ_2_-F_1_	3.4	(0.18, 0.17)	6.0	7.77	6.46	14 143	4.2	5.42	2.54

^*a*^Turn on voltage at a brightness of 1 cd m^–2^.

^*b*^1 mA cm^–2^.

^*c*^External quantum efficiency.

^*d*^Maximum current efficiency.

^*e*^Maximum power efficiency.

^*f*^Maximum luminance.

As shown in Fig. 2b, the devices based on **1** (7 wt%) in both CBZ_2_-F_1_ and TBCPF exhibit the more balanced dual-emission property, in which the ratio of enol-form emission is larger than that in the PL spectrum of **1** in toluene illustrated in Fig. 1. The solid film of **1** (7 wt%) in TBCPF also shows a typical yellowish-green emission with CIE coordinates of (0.30, 0.51), in which the ratio of enol-form emission is even less than that in toluene (Fig. 1 and 2b). In the EL spectrum, the intensity of the enol-form emission of **1** (7 wt%) in TBCPF (0.59) is approximately three times that in the PL spectrum (0.22). The thin film of **2** (2 wt%) in CBZ_2_-F_1_ exhibits blue-white light emission with CIE coordinates of (0.24, 0.23) (Fig. 2c). The PL spectrum of **2** in toluene presents a fine balance between the enol-form emission and the keto-form emission, displaying a nearly pure white light emission with CIE coordinates of (0.27, 0.33) (Fig. 1). In its EL spectrum, the enol-form emission is increased significantly, and the keto-form emission is weakened remarkably. Therefore, the device based on **2** (2 wt%) in CBZ_2_-F_1_ exhibits sky-blue emission with CIE coordinates of (0.18, 0.16) (Fig. 2c).

### Electroluminescence mechanism

To get some insight into the ratio change of the dual-emission in the PL and EL spectra, the electroluminescence mechanism was investigated. The singlet exciton yields of the EL devices were calculated according to eqn (1):^5^,^8^

1 *η*_s_ = EQE_max_/(*γ* × *η*_PL_ × *η*_out_)

Where *η*_s_ is the singlet exciton yield; EQE_max_ is the maximum external quantum efficiency; *η*_out_ is the light-out-coupling efficiency (about 20%); *η*_PL_ is the PL quantum efficiency; and *γ* is the electron–hole recombination probability in the emission layer, which is assumed as 100%. Thus, the *η*_s_ values of the devices based on **1** (7%) in TBCPF and **2** (2%) in CBZ_2_-F_1_ were calculated to be 63% and 91%, respectively, which exclude the possibility of the EL mechanism being triplet–triplet annihilation (TTA),^5^,^9^ because the *η*_s_ should not exceed its maximum percentage of 62.5% in the TTA mechanism.^9^

Subsequently, the thermally activated delayed fluorescence (TADF) molecule 4,4′-(phenazine-5,10-diyl)dibenzonitrile (DHPZ-2BN) was synthesized as a reference compound.^10^ The transient PL spectra of **1**, **2** and DHPZ-2BN in doped films were measured at room temperature. As shown in Fig. S10,† the TADF material DHPZ-2BN shows relatively flat PL decay. By comparison, **1** and **2** exhibit very sharp PL decay curves, indicating that the radiative excitons in **1** and **2** are short-lived components without TADF characteristics.^11^ Meanwhile, oxygen quenching experiments were performed (Section VIII, ESI†). In N_2_-degassed toluene, the emission band of DHPZ-2BN displays a double-exponential decay (*τ*_1_ = 6.79 ns, 25%; *τ*_2_ = 1891 ns, 75%). Upon bubbling with air, its PL intensity decreases rapidly, together with a dramatically shortened decay lifetime (*τ*_1_ = 4.92 ns, 94%; *τ*_2_ = 25.8 ns, 6%) (Table S4, Fig. S11 and S12†). By contrast, the transient PL spectra of **1** and **2** do not exhibit lifetime components in N_2_-degassed toluene, and oxygen quenching effects are not obvious either (Table S4, Fig. S11 and S12†).^11^ A possible reason is that the ultrafast RISC from the triplet state to the singlet state leads to insufficient triplet exciton accumulation, which is consistent with the common characteristic of organic EL materials with HLCT excited state character.^5^

**Fig. 3:**
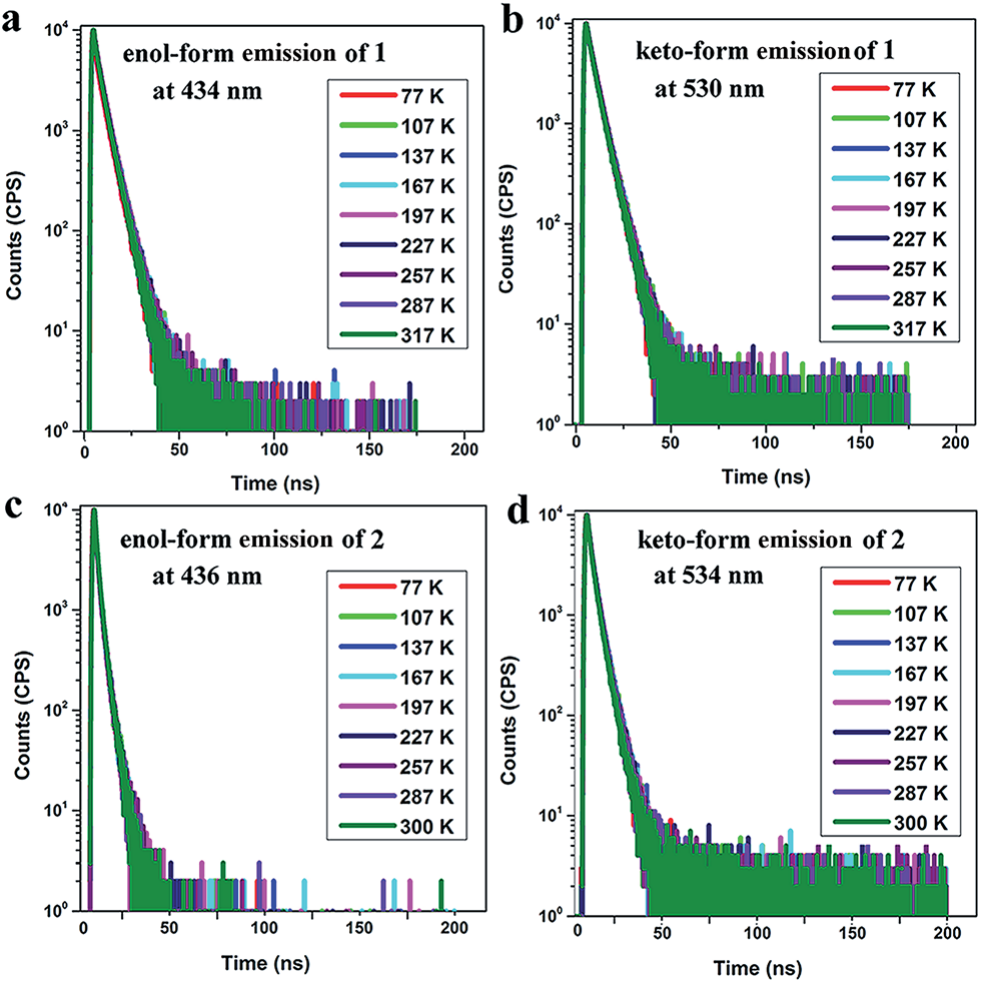
Temperature-dependent transient emission spectra ((a) enol-form emission at 434 nm; (b) keto-form emission at 530 nm) of a doped film (7 wt% **1** in TBCPF); and temperature-dependent transient emission spectra ((c) enol-form emission at 436 nm; (d) keto-form emission at 534 nm) of a doped film (2 wt% **2** in CBZ_2_-F_1_).

Furthermore, the room temperature PL lifetimes of **1** and **2** in other solvents were investigated and the results are summarized in Tables S5 and S6.† It is observed that there are several lifetime components with nanosecond order and no delayed fluorescence components exist in both the enol-form and keto-form emissions. The temperature-dependent PL measurements for the thin films of **1** (7 wt%) in TBCPF and **2** (2 wt%) in CBZ_2_-F_1_ also show no delayed lifetime components in both the enol-form and keto-form emissions with the temperature increase from 77 K to 300 K or 317 K (Fig. 3, Tables S7 and S8†). Thus, we deduced that both of the emitters (**1** and **2**) may possess the HLCT excited state character rather than the TADF mechanism.

**Fig. 4:**
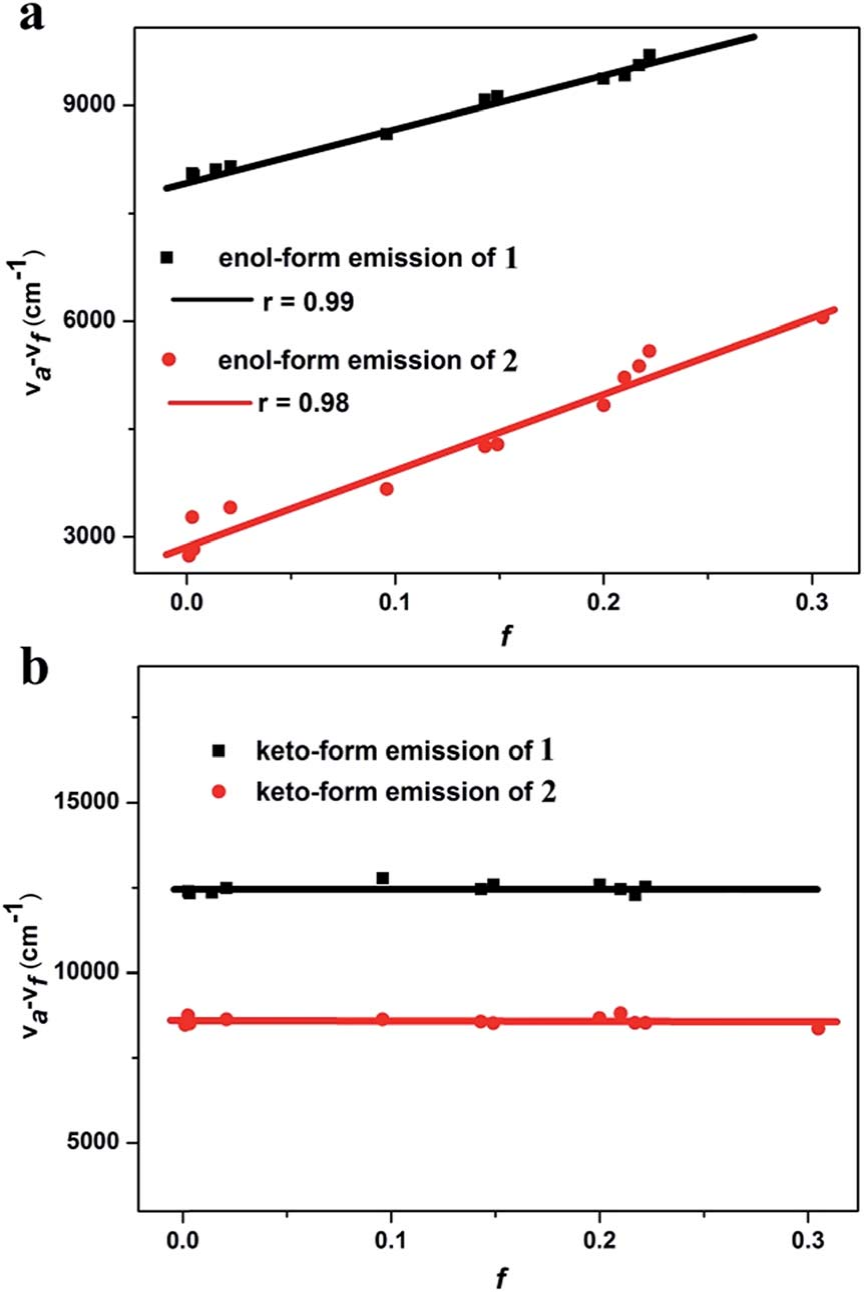
Linear fitting of the Lippert–Mataga model ((a) enol-form emission; and (b) keto-form emission. *f*: orientation polarization of the solvent media; and *ν*_a_ – *ν*_f_: Stokes shift of the enol-form and keto-form of **1** and **2** in different solvents).

To confirm the HLCT state character of **1** and **2**, the solvation effects on absorption and emission were studied in different solvents (Fig. 4 and Section XI, ESI†).^5^ As shown in Fig. S13, and Tables S9 and S10,† the absorption spectra of **1** and **2** exhibit only a slight change in absorption shape and peak position with an increase of solvent polarity. However, the enol-form emissions of **1** and **2** are remarkably affected by the solvent polarity, whereas their keto-form emissions are nearly solvent-independent. The enol-form emission peak of **1** exhibits an obvious red shift from 429 nm in nonpolar cyclohexane to 464 nm in polar 1,2-dichloroethane. For the emitter **2**, a larger red shift up to 68 nm is observed with the change of solvent polarity from the low-polarity cyclohexane to the high-polarity acetonitrile. The large solvatochromic shift indicates that the enol-form emissions of **1** and **2** are of typical ICT character (Fig. 4, Tables S9 and S10†).^5^,^12^–^14^

The solvent polarity-dependent emission properties of **1** and **2** were studied using the Lippert–Mataga model.^12^ As shown in Fig. 4a, the enol-form emissions of **1** and **2** display good linear correlations between the Stokes shift (*ν*_a_ – *ν*_f_) and solvent polarity parameter (*f*), which show only one slope value of 7008 (*r* = 0.99) and 10 577 (*r* = 0.98) for **1** and **2**, respectively (Fig. 4). The dipole moment changes of the enol-forms of **1** and **2** between the excited state and the ground state (Δ*μ* = *μ*_e_ – *μ*_g_), which can serve as an empirical scale to evaluate the strength of the ICT,^13^ were calculated to be 8.0 D and 15.2 D, respectively (Section XI, ESI†). These observations clearly illustrate that the enol-forms of **1** and **2** in the excited state possess the ICT character.^5^,^13^ However, linear relationships with slopes of near zero for various solvents are drawn for the keto-forms of **1** and **2**, indicating no obvious dipole moment changes for the keto-forms of **1** and **2** from the ground state to the excited state. These results reveal that the keto-forms of **1** and **2** are not of the distinct ICT character (Fig. 4b, Tables S9 and S10†).

As shown in Fig. S13c and d,† in the PL spectra the enol-form emissions of both **1** and **2** exhibit vibrational fine structure in low polar solvents such as cyclohexane and butyl ether, which reveals the existence of the LE state for the enol-forms of **1** and **2** (Section XI, ESI†).^5^ In contrast, the keto-form emissions of **1** and **2** do not show the vibrational fine structure in various polar solvents. The excited state dipole moments (*μ*_e_) of the enol-forms of **1** and **2** were calculated to be 11.9 D and 18.9 D, respectively, which are between those of a usual LE emitter (*ca.* 8 D) and a typical CT molecule such as 4-(*N*,*N*-dimethylamino)benzonitrile (*μ*_e_ = 23 D).^5^,^14^ These results indicate that the excited state enol-forms of **1** and **2** possess the typical character of HLCT, while their excited state keto-forms are not of the HLCT character.^5^

**Fig. 5:**
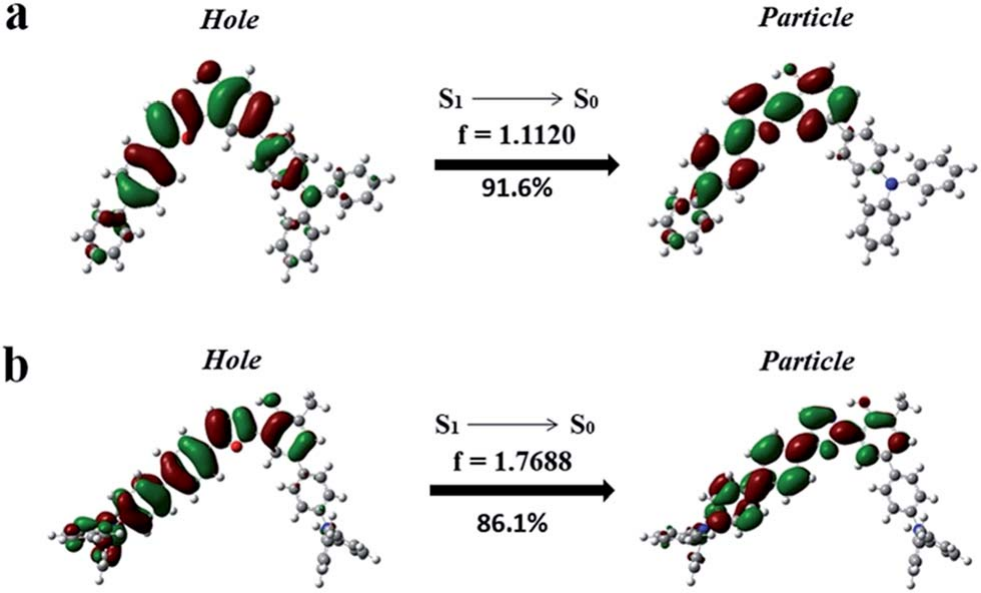
Natural transition orbitals (NTOs) of S_1_ → S_0_ of the enol-forms of **1** (a) and **2** (b).

To further confirm the HLCT excited state character of the enol-forms of **1** and **2**, the natural transition orbitals (NTOs) of S_1_ → S_0_ of the enol-forms of **1** and **2** were calculated using the TD-M06-2X/6-311+G(d, p) method (Section XII, ESI†).^5^ As shown in Fig. 5, the particle of **1** is mainly distributed on the phenol ring and 5-biphenyl oxazole, while the hole is spread over the whole molecule skeleton, except for the two outstretched phenyl rings of TPA on the side of the phenol. The particle and hole distributions are almost completely overlapped from the phenol ring to the 5-biphenyl oxazole, demonstrating the LE characterized transition from S_1_ to S_0_, whereas the particle and hole distributions do not overlap on the whole TPA skeleton, indicating a CT characterized transition from S_1_ to S_0_. As shown in Fig. 5b, for the enol-form of **2**, the overlapped region of the particle and hole distributions is also from the phenol ring to the 5-biphenyl oxazole, which is almost the same as that of **1**. The separated region of the particle and hole distributions of **2** mainly involves the TPAs on both sides of the molecule. The particle and hole NTOs for both **1** and **2** exhibit a balance between spatial separation and orbital overlap. The separated orbital favors CT character, while the moderate orbital overlap favors LE character.^5^

**Fig. 6:**
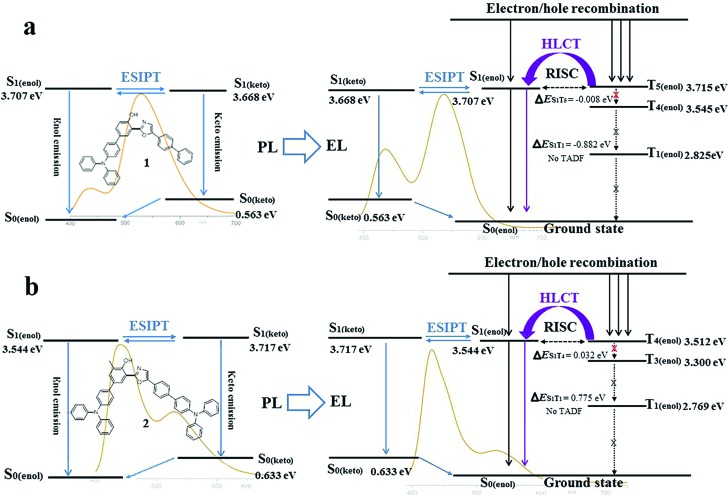
Diagrams of the calculated energy levels of **1** (a) and **2** (b) in PL processes and the simplified model for exciton relaxation in EL processes. Background: PL and EL spectra of **1** (7 wt%) in TBCPF and **2** (2%) in CBZ_2_-F_1_.

Subsequently, the energy levels of the singlet and triplet excited states of **1** and **2** were calculated using TDDFT and the corresponding energy diagrams are shown in Fig. 6 (Tables S11 and S12†).^5^ For the enol-form of **1**, the excited state energies of S_1(enol)_, T_1(enol)_, T_2(enol)_, T_3(enol)_, T_4(enol)_ and T_5(enol)_ were calculated to be 3.707, 2.825, 3.237, 3.415, 3.545 and 3.715 eV, respectively. The large energy gap between S_1_ and T_1_ reaches 0.882 eV, which indicates that it is almost impossible for the RISC from T_1(enol)_ to S_1(enol)_ to take place. Thus, the thermally activated delayed fluorescence (TADF) model could be excluded.^11^ The excited state energy levels of S_1(enol)_ and T_5(enol)_ are almost identical, indicating a potential RISC process from T_5(enol)_ to S_1(enol)_. Moreover, the energy gap between T_5(enol)_ and T_4(enol)_ (0.170 eV) is larger than that between T_5(enol)_ and S_1(enol)_ (0.008 eV), indicating that the internal conversion (IC) rate from T_5(enol)_ to T_4(enol)_ may be lower than the RISC rate from T_5(enol)_ to S_1(enol)_.^3^ Thus, it could be inferred that the enol-form of **1** may indeed possess the HLCT excited state in the EL process. In addition, the excited state energy level of the keto-form of **1** (S_1(keto)_) was calculated to be 3.668 eV, which is close to that of the enol-form of **1** (S_1(enol)_), demonstrating the existence of the thermodynamic equilibrium between the excited state enol-form and keto-form through the ESIPT reaction, and therefore, the dual-emission of both the enol-form and keto-form could be observed.2d,g,^3^,^4^ Accordingly, the electroluminescence of **1** originates from a synergistic combination of a HLCT excited state character-induced RISC process and an efficient ESIPT process. For the enol-form of **2**, the energy level of S_1(enol)_ (3.544 eV) is close to that of T_4(enol)_ (3.512 eV), indicating that RISC from T_4(enol)_ to S_1(enol)_ may take place. The excited state energy levels of the enol-form and keto-form of **2** are also close to each other, illustrating the existence of the ESIPT equilibrium. Therefore, the electroluminescence of **2** still consists of an RISC process and a synergistic ESIPT reaction.

Despite the existence of the ESIPT equilibrium between the enol-form and the keto-form, the energy level of the enol-form of **1** (S_1(enol)_) is slightly higher than that of its keto-form (S_1(keto)_), demonstrating that this equilibrium prefers to form the excited state keto-form rather than the enol-form. Therefore, in the PL spectrum of **1** (7 wt%) in TBCPF, the proportion of the keto-form emission is much greater than that of the enol-form emission. When the intensity of the keto-form emission is normalized to 1.00, the intensity of the enol-form emission is only 0.22 (Fig. 2b). However, in the EL spectrum of the device based on **1** (7 wt%) in TBCPF, the triplet excitons of the enol-form **1** can continuously transfer to the enol-form singlet excitons through the RISC from T_5(enol)_ to S_1(enol)_, leading to an increase in the number of enol-form singlet excitons. Then, the ESIPT equilibrium is re-established towards an enhanced enol-form emission. Thus, the intensity of the enol-form emission of the device based on **1** (7 wt%) in TBCPF reaches 0.59, which is almost three times that in the PL spectrum (Fig. 2b). Different from the enol-form of **1**, the energy level of the enol-form of **2** (S_1(enol)_) is slightly lower than that of the keto-form of **2** (S_1(keto)_), indicating that the ESIPT equilibrium is in favor of the enol-form.^1^ Thus, in the PL spectrum of **2** (2 wt%) in CBZ_2_-F_1_, the proportion of the keto-form emission is less than that of the enol-form emission. When the intensity of the enol-form emission is normalized to 1.00, the intensity of the keto-form emission is 0.49 (Fig. 2c). Similarly, under electric field excitation, the number of enol-form singlet excitons of **2** is further increased due to the RISC from T_4(enol)_ to S_1(enol)_, and then the ESIPT equilibrium is re-established towards the direction more favorable to the enol-form emission. Thus, the intensity of the keto-form emission of the device based on **2** (2 wt%) in CBZ_2_-F_1_ drops to 0.21 (Fig. 2c). Briefly, the RISC of the enol-form excitons from the triplet state to the singlet state triggers an increase in enol-form singlet excitons, which further leads to a shift of the ESIPT equilibrium towards an enhanced enol-form fluorescence emission. Thus, the difference between the ESIPT equilibria in photoluminescence and electroluminescence may be ascribed to the HLCT character of the enol-form excited state.

## Conclusions

In summary, the photoluminescence and electroluminescence of two highly efficient ESIPT molecules, 2-(2′-hydroxyphenyl)oxazoles containing one TPA (**1**) and two TPAs (**2**) respectively, are investigated systematically. Experimental data and theoretical calculations demonstrate that the enol-forms of both **1** and **2** possess the HLCT excited state character, while their excited state keto-forms are not of the obvious HLCT character. The electroluminescence of **1** and **2** originates from the synergistic combination of the ESIPT equilibrium and the RISC process arising from the HLCT excited state character. Owing to the RISC of the enol-form excitons of **1** from the triplet state to the singlet state in the electroluminescence, the ESIPT equilibrium is re-established towards an enhanced enol-form fluorescence emission. Therefore, the fluorescence intensity of the enol-form **1** in the EL spectrum (0.59) is approximately three times that in the PL spectrum (0.22). The maximum EQE of the device based on **1** (7 wt%) in TBCPF reaches 5.3%, which is the highest EQE value recorded for single molecular white light-emitting materials. The fluorescence intensity of the enol-form **2** in the EL spectrum is also increased remarkably relative to its PL spectrum due to the RISC of the enol-form excitons of **2**. Therefore, the device based on **2** (2 wt%) in CBZ_2_-F_1_ shows sky-blue emission with CIE coordinates of (0.18, 0.16) and an EQE of 8.0%, which is the highest EQE in the reported HLCT materials. The discovery of an ESIPT equilibrium with an RISC process would offer new insight into the design of EL devices based on ESIPT emitters.

## Conflicts of interest

There are no conflicts to declare.

## Supplementary Material

Supplementary information
