# Supplementary material for: Dual-emissive 2-(2′-hydroxyphenyl)oxazoles for high performance organic electroluminescent devices: discovery of a new equilibrium of excited state intramolecular proton transfer with a reverse intersystem crossing process
Source: Chem Sci. 2017 Dec 1;9(5):1213–20. doi: 10.1039/c7sc04464j (PMC5885805; doi:10.1039/c7sc04464j)
Supplement: Supplementary file 1 [file SC-009-C7SC04464J-s001.pdf]

Electronic Supplementary Material (ESI) for Chemical Science.
This journal is © The Royal Society of Chemistry 2017

Supporting Information

Dual-emissive 2-(2'-Hydroxyphenyl)oxazoles for high performance organic electroluminescent devices: discovery of a new equilibrium of excited state intramolecular proton transfer with a reverse intersystem crossing process

Bijin Li,[‡a] Linsen Zhou,[‡c] Hu Cheng,[a] Quan Huang,[a] Jingbo Lan,*[a] Liang Zhou,*[b] and Jingsong You*[a]

[a]*Key Laboratory of Green Chemistry and Technology of Ministry of Education, College of Chemistry, Sichuan University, 29 Wangjiang Road, Chengdu 610064, PR China*

E-mail address: jsyou@scu.edu.cn; jingbolan@scu.edu.cn

[b]*State Key Laboratory of Rare Earth Resource Utilization, Changchun Institute of Applied Chemistry, Chinese Academy of Sciences, 5625 Renmin Street, Changchun 130022, PR China*

E-mail: zhoul@ciac.ac.cn.

[c]*Center of Interface Dynamics for Sustainability, Institute of Materials, China Academy of Engineering Physics, 596 Yinhe Road, Chengdu 610200, PR China*

[‡] *These authors contributed equally to this work.*

I. General remarks

NMR spectra were recorded with a Bruker AV II-400 spectrometer. The ^{1}H NMR (400 MHz) chemical shifts were measured relative to $CDCl_3$ or DMSO-d_6 as the internal reference (DMSO-d_6: δ 2.50 ppm; $CDCl_3$: δ 7.26 ppm). The ^{13}C NMR (100 MHz) chemical shifts were given using DMSO-d_6/$CDCl_3$ as the internal standard (DMSO-d_6: δ 39.52 ppm; $CDCl_3$: δ 77.16 ppm). High resolution mass spectra (HR-MS) were performed on ESI-TOF. Melting points were recorded with XRC-1 and are uncorrected. UV−vis spectra were recorded using a HITACHI U-2910 spectrometer. Fluorescence spectra and absolute quantum yields were taken using a Horiba Jobin Yvon-Edison Fluoromax-4 fluorescence spectrometer with a calibrated integrating sphere system. The excited state lifetimes were obtained using an HORIBA TEMPRO-01 instrument. Cyclic voltammetry (CV) measurement was performed on LK2010A using an Ag/Ag^+ reference electrode, a platinum wire counter electrode, and a platinum plate working electrode. Energy levels were calculated with respect to a standard ferrocene/ferrocenium (Fc/Fc^+) redox couple. Differential scanning calorimetry (DSC) data was recorded using a TA instrument DSC-Q200 1474. The decomposition temperature corresponding to 5% weight loss was conducted on NETZSCH TG 209F1 Iris.

Unless otherwise noted, chemicals and reagents were purchased from commercial suppliers without further purification. All syntheses and manipulations were performed under N_2 atmosphere in a glove box or using standard Schlenk. Solvents were dried by refluxing for at least 12 h over CaH_2 (DMF and DCM), sodium (1,4-dioxane, THF and toluene), magnesium and iodine (CH_3OH), and freshly distilled and vigorously purged with N_2 for 1 hour prior to use. *O*-Mesitylsulfonylhydroxylamine was prepared according to the literature procedures.[1] 4,4'-(Phenazine-5,10-diyl)dibenzonitrile (DHPZ-2BN) was synthesized according to the literature procedure.[2]

II. Device fabrication

Indium−tin−oxide (ITO) coated glass with a sheet resistance of 10 Ω sq^{-1} was used as the anode substrate. Prior to film deposition, patterned ITO substrates were cleaned with detergent, rinsed in deionized water, dried in an oven, and finally treated with oxygen plasma for 5 min at a pressure of 10 Pa to enhance the surface work function of ITO anode (from 4.7 to 5.1 eV). All of the organic layers were deposited with the rate of 0.1 nm s^{-1} under high vacuum (≤ 2×10^{-5} Pa). The doped and co-doped layers were prepared by co-evaporating dopant(s) and host material from two or three individual sources, and the doping concentrations were modulated by controlling the evaporation rates of dopant(s). MoO_3, LiF, and Al were deposited in another vacuum chamber (≤ 8.0×10^{-5} Pa) with the rates of 0.01 and 1 nm s^{-1}, respectively, without being exposed to the atmosphere. The thicknesses of these deposited layers and the evaporation rate of individual materials were monitored in vacuum with quartz crystal monitors. A shadow mask was used to define the cathode and to make ten devices (10 mm^2) on each substrate.

Measurements: Current density−voltage−Luminance (*J*−*V*−*L*) characteristics were measured by using a programmable Keithley source measurement unit (Keithley 2400 and Keithley 2000) with a silicon photodiode. The EL spectra were measured with a calibrated Hitachi F−7000 fluorescence spectrophotometer. On the basis of the uncorrected EL fluorescence spectra, the Commission Internationale de l'Eclairage (CIE) coordinates were calculated using the test program of Spectrascan PR650 spectrophotometer. The EQE of EL devices were calculated based on the photo energy measured by the photodiode, the EL spectrum, and the current pass through the device.

III. Synthesis of 2-(2'-hydroxyphenyl)oxazoles

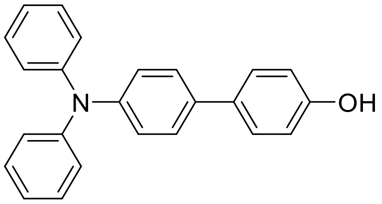

4'-(Diphenylamino)biphenyl-4-ol (5a)

Compund **5a** was synthesized according to the literature procedure.[3b] To a dried 100 mL schlenk tube with a magnetic stirring bar were added $Pd(OAc)_2$ (16.9 mg, 75 μmol), XPhos (71.5 mg, 0.15 mmol), 4-iodophenol (220.0 mg, 1.0 mmol), 4-(diphenylamino)phenylboronic acid (578.0 mg, 2.0 mmol), K_3PO_4 (636.6 mg, 3.0 mmol) and THF (4.0 mL) under N_2 atmosphere. The reaction mixture was then stirred at 80 °C for 12 h. After being cooled to room temperature, the reaction mixture was diluted with 10-20 mL of EtOAc, filtered through a celite pad, and washed with 10-20 mL of EtOAc. Organic solvent was removed under reduced pressure. The residue was purified by flash column chromatography (petroleum ether/ethyl acetate = 6/1, v/v) to afford the desired product as an off-white solid (320 mg, 95%). M.p.: 132-134 °C; ^{1}H NMR (400 MHz, DMSO-d_6): δ (ppm) 9.49 (s, 1H), 7.49 (d, J = 8.4 Hz, 2H), 7.44 (d, J = 8.4 Hz, 2H), 7.31-7.27 (m, 4H), 7.05-7.00 (m, 8H), 6.82 (d, J = 8.4 Hz, 2H); ^{13}C NMR (100 MHz, DMSO-d_6): δ (ppm) 156.8, 147.2, 145.7, 134.7, 130.4, 129.5, 127.3, 126.9, 124.0, 123.7, 122.9, 115.7; HRMS (ESI$^+$): calcd for $C_{24}H_{20}NO$ $[M+H]^+$ 338.1545, found 338.1549.

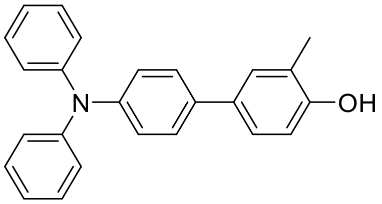

4'-(Diphenylamino)-3-methylbiphenyl-4-ol (5b)

Compund **5b** was synthesized according to the literature procedure.[3b] To a reaction tube with a magnetic stirring bar, $Pd(OAc)_2$ (7.5 mol%, 16.9 mg, 75 μmol), XPhos (15 mol%, 71.5 mg, 0.15 mmol), 4-bromo-2-methylphenol (187.0 mg, 1.0 mmol), 4-(diphenylamino)phenylboronic acid (578.0 mg, 2.0 mmol), K_3PO_4 (636.6 mg, 3.0 mmol) and THF (4.0 mL) were added under N_2 atmosphere. The reaction mixture was stirred at 80 °C for 12 h. After being cooled to room temperature, the reaction mixture was diluted with EtOAc (10-20 mL), filtered through a pad of celite, and then the solvent of filtrate was removed under reduced pressure. The residue was purified by flash column chromatography (petroleum ether/ethyl acetate = 6/1, v/v) to afford the desired product as an off-white solid (317 mg, 90%). M.p.: 112-114 °C; ^{1}H NMR (400 MHz, DMSO-d_6): δ (ppm) 9.37 (s, 1H), 7.48 (d, J = 8.4 Hz, 2H), 7.34-7.24 (m, 6H), 7.04-6.99 (m, 8H), 6.83 (d, J = 8.4 Hz, 1H), 2.17 (s, 3H); ^{13}C NMR (100 MHz, DMSO-d_6): δ (ppm) 154.9, 147.2, 145.5, 135.0, 130.3, 129.5, 128.5, 126.9, 124.5, 124.2, 124.0, 123.6, 122.8, 115.0, 16.1; HRMS (ESI$^+$): calcd for $C_{25}H_{22}NO$ $[M+H]^+$ 352.1701, found 352.1694.

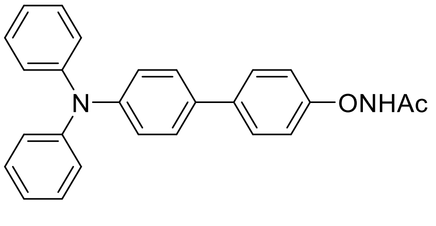

***N*-(4'-(Diphenylamino)biphenyl-4-yloxy)acetamide (6a)**

The synthesis of **6a** was followed by the procedure described in previous method.[3] 4'-(Diphenylamino)biphenyl-4-ol (800 mg, 2.37 mmol) was dissolved in 7 mL of methanol, and then potassium *tert*-butoxide (267 mg, 2.37 mmol) was added. The mixture was allowed to stir for 0.5 h under N_2 atmosphere. Methanol was removed, and the residue was taken up in 5 mL of dichloromethane. The freshly prepared *O*-mesitylsulfonylhydroxylamine (380 mg, 1.77 mmol) in 5 mL of dichloromethane was then added under ice cooling. The mixture was

allowed to stir for 1 h, and dichloromethane was then removed under reduce pressure to afford 4'-(aminooxy)-*N*,*N*-diphenyl-[1,1'-biphenyl]-4-amine.

A biphasic mixture of Na_2CO_3 (190 mg, 1.8 mmol) in 1 mL of H_2O and 2 mL of EtOAc was next added to the reaction flask. The resulting solution was kept under ice cooling, followed by dropwise addition of acetyl chloride (138.9 mg, 1.77 mmol). After being stirred at 0 ^oC for 2 h, the reaction was quenched with sat. $NaHCO_3$ and diluted with EtOAc. The organic phase was washed twice with sat. $NaHCO_3$, dried over Na_2SO_4, filtered, and evaporated under reduced pressure. The residue was purified by flash column chromatography (CH_2Cl_2/ethyl acetate = 8/1-4/1, v/v) afforded the desired product as a white solid (446 mg, 64%) with the recovery of 28% of unreacted 4'-(diphenylamino)biphenyl-4-ol (220 mg, 28%). M.p.: 148-150 ^oC; ^1H NMR (400 MHz, DMSO-d_6): δ (ppm) 11.71 (s, 1H), 7.58 (d, J = 8.8 Hz, 2H), 7.54 (d, J = 8.4 Hz, 2H), 7.31 (t, J = 8.4 Hz, 4H), 7.07-7.02 (m, 10H), 1.93 (s, 3H); ^{13}C NMR (100 MHz, DMSO-d_6): δ (ppm) 158.8, 147.1, 146.3, 133.8, 129.5, 127.3, 127.2, 123.9, 123.6, 123.0, 113.4, 19.4; HRMS (ESI$^+$): calcd for $C_{26}H_{23}N_2O_2$ $[M+H]^+$ 395.1760, found 395.1764.

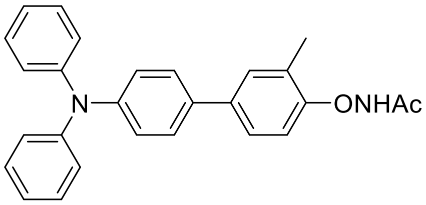

N-(4'-(Diphenylamino)-3-methylbiphenyl-4-yloxy)acetamide (6b)

The synthesis of **6b** was followed by the procedure described in previous method.[3] 4'-(Diphenylamino)-3-methylbiphenyl-4-ol (1.63 g, 4.64 mmol) was dissolved in 10 mL of methanol, and then potassium *tert*-butoxide (520 mg, 4.64 mmol) was added. The mixture was allowed to stir for 0.5 h under N_2 atmosphere. Methanol was removed, and the residue was taken up in 8 mL of dichloromethane. The freshly prepared *O*-

mesitylsulfonylhydroxylamine (572 mg, 2.67 mmol) in 8 mL of dichloromethane was then added under ice cooling. The mixture was allowed to stir for 1 h, and dichloromethane was then removed under reduce pressure to afford 4'-(aminooxy)-3'-methyl-*N*,*N*-diphenyl-[1,1'-biphenyl]-4-amine.

A biphasic mixture of Na_2CO_3 (817 mg, 2.67 mmol) in 1 mL of H_2O and 2 mL of EtOAc was next added to the reaction flask. The resulting solution was kept under ice cooling, followed by dropwise addition of acetyl chloride (209 mg, 2.66 mmol). After being stirred at 0 °C for 2 h, the reaction was quenched with sat. $NaHCO_3$ and diluted with EtOAc. The organic phase was washed twice with sat. $NaHCO_3$, dried over Na_2SO_4, filtered, and evaporated under reduced pressure. The residue was purified *via* silica gel column chromatography (CH_2Cl_2/ethyl acetate = 8/1-4/1, v/v) afforded the desired product as a white solid (490 mg, 45%) with the recovery of 34% of unreacted 4'-(diphenylamino)-3-methylbiphenyl-4-ol (560 mg, 34%). M.p.: 126-128 °C; ^{1}H NMR (400 MHz, DMSO-*d*$_6$): *δ* (ppm) 11.70 (s, 1H), 7.53 (d, *J* = 8.4 Hz, 2H), 7.44 (s, 1H), 7.40 (d, *J* = 8.4 Hz, 1H), 7.31 (t, *J* = 7.6 Hz, 3H), 7.06-7.02 (m, 10H), 2.26 (s, 3H), 1.93 (s, 3H); ^{13}C NMR (100 MHz, DMSO-*d*$_6$): *δ* (ppm) 167.1, 156.8, 147.1, 146.2, 134.0, 133.6, 129.5, 128.6, 127.3, 124.6, 123.9, 123.8, 123.7, 123.0, 112.1, 19.4, 15.6; HRMS (ESI$^+$): calcd for $C_{27}H_{24}N_2O_2Na$ $[M+Na]^+$ 431.1735, found 431.1737.

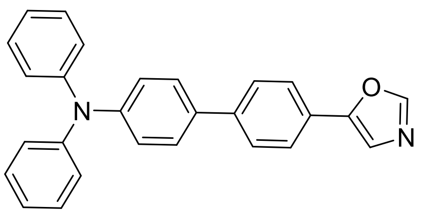

4'-(Oxazol-5-yl)-*N,N*-diphenyl-[1,1'-biphenyl]-4-amine (8)

Compund **8** was synthesized according to the literature procedure.[3b] To a reaction tube with a magnetic stirring bar, $Pd(OAc)_2$ (5 mol%, 11.3 mg, 50 µmol), XPhos (10 mol%, 47.7 mg, 0.1

mmol), 5-(4'-bromophenyl)oxazole (223.0 mg, 1.0 mmol) and 4-(diphenylamino)phenylboronic acid (578.0 mg, 2.0 mmol), K_3PO_4 (636.6 mg, 3.0 mmol) and THF (4.0 mL) were added under N_2 atmosphere. The reaction mixture was stirred at 80 °C for 12 h. The reaction mixture was then cooled to ambient temperature, diluted with 10-20 mL of EtOAc, filtered through a celite pad, and washed with 10-20 mL of EtOAc. Organic solvent was removed under reduced pressure. The residue was purified by flash column chromatography (petroleum ether/ethyl acetate = 7/1, v/v) to afford the desired product as a pale yellow solid (365 mg, 94%). M.p.: 138-140 °C; ^{1}H NMR (400 MHz, $CDCl_3$): *δ* (ppm) 7.93 (s, 1H), 7.71 (d, *J* = 8.4 Hz, 2H), 7.64 (d, *J* = 8.4 Hz, 2H), 7.50 (d, *J* = 8.8 Hz, 2H), 7.38 (s, 1H), 7.30-7.26 (m, 4H), 7.16-7.14 (m, 6H), 7.06 (t, *J* = 7.2 Hz, 2H); ^{13}C NMR (100 MHz, $CDCl_3$): *δ* (ppm) 151.6, 150.5, 147.8, 147.7, 141.0, 134.0, 129.5, 127.7, 127.1, 126.3, 125.0, 124.7, 123.8, 123.3, 121.5; HRMS (ESI$^+$): calcd for $C_{27}H_{21}N_2O$ $[M+H]^+$ 389.1654, found 389.1651.

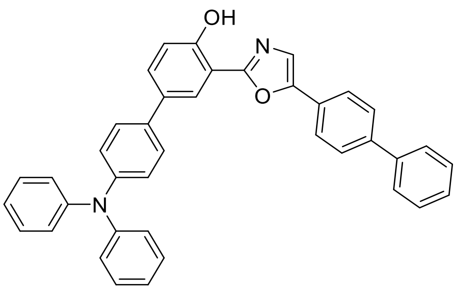

3-(5-([1,1'-Biphenyl]-4-yl)oxazol-2-yl)-4'-(diphenylamino)-[1,1'-biphenyl]-4-ol (1)

A flame-dried Schlenk test tube with a magnetic stir bar was charged with **6a** (157.6 mg, 0.4 mmol), 5-([1,1'-biphenyl]-4-yl)oxazole (132.6 mg, 0.6 mmol), $[RhCp^*Cl_2]_2$ (5.0 mol%, 12.8 mg), $AgSbF_6$ (20 mol%, 27.2 mg), PivOH (82.0 mg, 0.8 mmol), CsOPiv (76.0 mg, 0.32 mmol), Ag_2CO_3 (44.0 mg, 0.16 mmol) and DMF (3.0 mL) under N_2 atmosphere. The resulting mixture was stirred for 5 min at room temperature, and then heated at 140 °C for 24 h. The reaction mixture was cooled to ambient temperature, and diluted with EtOAc. The mixture was filtered through a celite pad and washed with EtOAc. The organic phase was then washed

with brine, dried over Na_2SO_4, and evaporated under vacuum. The residue was purified by flash column chromatography (petroleum ether/ethyl acetate = 7/1, v/v) afforded the desired product as a pale yellow solid (110 mg, 49%). M.p.: 220-222 °C; ^{1}H NMR (400 MHz, $CDCl_3$): δ (ppm) 11.20 (s, 1 H), 8.13 (d, J = 2.0 Hz, 1H), 7.82 (d, J = 8.0 Hz, 2H), 7.70 (d, J = 8.0 Hz, 2H), 7.64 (d, J = 7.6 Hz, 2H), 7.60 (dd, J = 8.4 Hz, 1.6 Hz, 1H), 7.53-7.46 (m, 5H), 7.39 (t, J = 7.2 Hz, 1H), 7.29 (t, J = 8.0 Hz, 4H), 7.19-7.15 (m, 7H), 7.05 (t, J = 7.2 Hz, 2H); ^{13}C NMR (100 MHz, $CDCl_3$): δ (ppm) 161.0, 156.8, 150.2, 147.9, 147.1, 141.8, 140.3, 134.6, 132.6, 131.0, 129.4, 129.1, 127.9, 127.8, 127.6, 127.1, 126.4, 125.0, 124.5, 124.3, 123.8, 123.1, 121.5, 117.8, 111.4; HRMS (ESI$^+$): calcd for $C_{39}H_{29}N_2O_2$ $[M+H]^+$ 557.2229, found 557.2229.

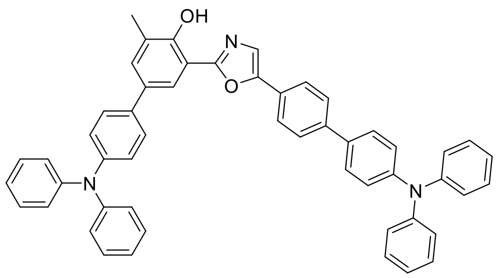

4'-(Diphenylamino)-3-(5-(4'-(diphenylamino)biphenyl-4-yl)oxazol-2-yl)-5-methylbiphenyl-4-ol (2)

A flame-dried Schlenk test tube with a magnetic stir bar was charged with **6b** (163.2 mg, 0.4 mmol), **8** (232.8 mg, 0.6 mmol), $[RhCp^*Cl_2]_2$ (5.0 mol%, 12.8 mg), $AgSbF_6$ (20 mol%, 27.2 mg), PivOH (82.0 mg, 0.8 mmol), CsOPiv (76.0 mg, 0.32 mmol), Ag_2CO_3 (44.0 mg, 0.16 mmol) and DMF (3.0 mL) under N_2 atmosphere. The resulting mixture was stirred for 5 min at room temperature, and then heated at 140 °C for 24 h. The reaction mixture was cooled to ambient temperature, and diluted with EtOAc. The mixture was filtered through a celite pad and washed with EtOAc. The organic phase was then washed with brine, dried over Na_2SO_4, and evaporated under vacuum. The residue was purified by flash column chromatography (petroleum ether/ethyl acetate = 7/1, v/v) afforded the desired product as a pale yellow

solid (132 mg, 45%). ^{1}H NMR (400 MHz, DMSO-d_6): δ (ppm) 11.31 (s, 1H), 8.05 (s, 1H), 8.00 (s, 1H), 7.95 (d, J = 7.6 Hz, 2H), 7.75 (d, J = 8.0 Hz, 2H), 7.64 (d, J = 8.4 Hz, 4H), 7.60 (s, 1H), 7.35-7.30 (m, 8H), 7.10-7.04 (m, 16H), 2.31 (s, 3H); ^{13}C NMR (100 MHz, $CDCl_3$): δ (ppm) 150.2, 147.9, 147.8, 147.7, 141.1, 137.9, 135.0, 133.9, 132.1, 131.0, 129.5, 129.4, 127.7, 127.6, 127.21, 127.17, 125.9, 124.9, 124.5, 124.4, 123.8, 123.3, 123.0, 121.5, 121.2, 110.8, 16.4; HRMS (ESI$^+$): calcd for $C_{52}H_{40}N_3O_2$ [M+H]$^+$ 738.3121, found 738.3114.

IV. Absorption spectra of 1 and 2 in toluene

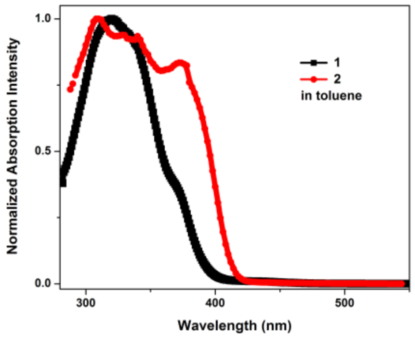

Fig. S1 Normalized absorption spectra of **1** and **2** in toluene (5.0×10^{-5} mol L^{-1}).

V. Thermal and electrochemistry properties of 1 and 2

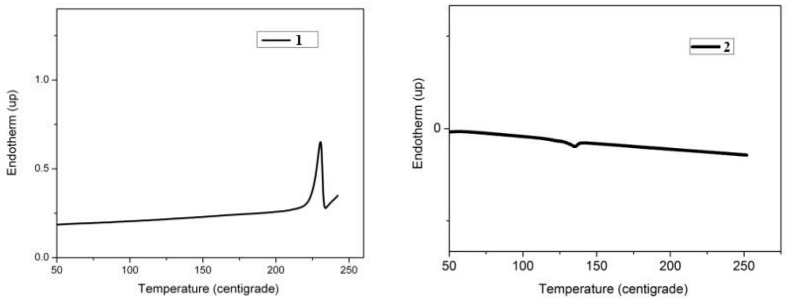

Fig. S2 Differential scanning calorimetry (DSC) graphs of **1** and **2**.

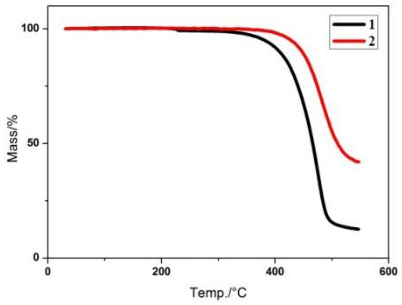

Fig. S3 Thermogravimetric analysis (TGA) curves of **1** and **2**.

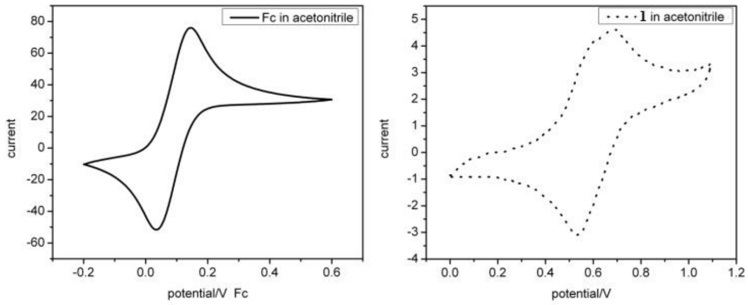

Fig. S4 The oxidation potential of **1** was determined relative to Ag/Ag^{+} in 5.0×10^{-4} mol L^{-1} acetonitrile solution, with the Fc/Fc^{+} as the external standard. Cyclic voltammogram of Fc/Fc^{+} in acetonitrile.

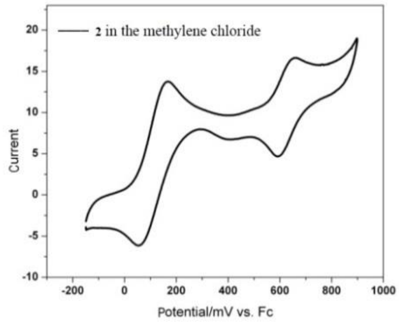

Fig. S5 Cyclic voltammogram of **2** in methylene chloride. The oxidation potentials were determined relative to Ag/Ag^{+} in 5.0×10^{-4} mol L^{-1} methylene chloride solution, using Fc/Fc^{+} as internal reference.

Table S1. The thermal and electrochemistry data of **1** and **2**.

Compounds	E_{g}^{opt}(eV)^{a}	E_{on}^{ox}(eV)	HOMO (eV)^{d}	LUMO (eV)^{e}	T_{d} [°C]^{f}
**1**	3.17	0.47^{b}	-5.27	-2.10	381
**2**	2.95	0.42^{c}	-5.22	-2.27	430

[a]$E_{\mathrm{g}}^{\mathrm{opt}}$ = 1240/λ_{onset} (eV). [b]Estimated from the oxidation onset of the cyclic voltammetry in 5.0 × 10^{-4} mol L^{-1} acetonitrile solution. [c]Estimated from the oxidation onset of the cyclic voltammetry in 5.0 × 10^{-4} mol L^{-1} CH_2Cl_2 solution. [d]HOMO = −(4.8 + $E_{\mathrm{on}}^{\mathrm{ox}}$) (eV). [e]LUMO = (HOMO + $E_{\mathrm{g}}^{\mathrm{opt}}$) (eV). [f]Temperature at 5.0% weight loss under nitrogen. The heating rate was 10 °C·min^{-1}.

VI. Device schematic and device structure

ITO = indium tin oxide, MoO_3 = molybdenum oxide, TAPC = 1,1-bis{(di-4-tolylamino)phenyl}cyclohexane, CBZ_2-F_1 = 3,3'-(9*H*-fluorene-9,9-diyl)bis(9-phenyl-9*H*-carbazole), TBCPF = 9,9-di(4,4'-bis(3,6-di-*tert*-butyl-9*H*-carbazole)-phenyl)-9*H*-fluorene, TmPyPB = 1,3,5-tri(*m*-pyrid-3-ylphenyl)benzene), Tm3PyP26PyB = 1,3,5-tris(6-(3-(pyridin-3-yl)phenyl)pyridin-2-yl)benzene, LiF = lithium fluoride, Al = aluminium.

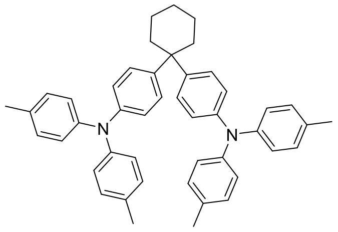

TAPC

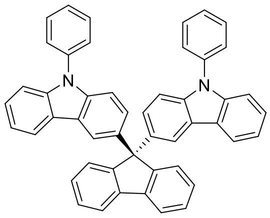

CBZ_2-F_1

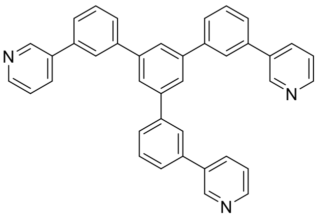

TmPyPB

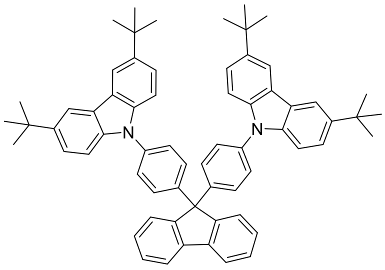

TBCPF

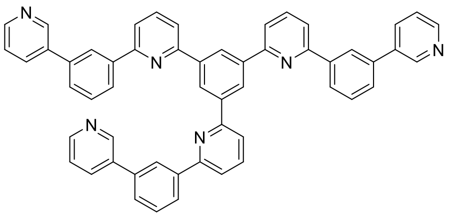

Tm3PyP26PyB

1-based devices:

1 (x%) in **CBZ_2-F_1:** ITO/MoO_3 (3 nm)/TAPC (50 nm)/**1** (x wt%): CBZ_2-F_1 (20 nm)/TmPyPB (50 nm)/LiF (1 nm)/Al (100 nm)

1 (x%) in **TBCPF:** ITO/MoO_3 (3 nm)/TAPC (50 nm)/**1** (x wt%): TBCPF (20 nm)/TmPyPB (50 nm)/LiF (1 nm)/Al (100 nm)

2-based devices:

2 (x%) in **CBZ_2-F_1:** ITO/MoO_3 (3 nm)/TAPC (50 nm)/**2** (x wt%):CBZ_2-F_1 (20 nm)/Tm3PyP26PyB (50 nm)/LiF (1 nm)/Al (100 nm)

2 (x%) in **TBCPF:** ITO/MoO_3 (3 nm)/TAPC (50 nm)/**2** (x wt%):TBCPF (20 nm)/Tm3PyP26PyB (50 nm)/LiF (1 nm)/Al (100 nm)

Table S2. Device performance with **1** and **2** as the emitter

Device	$V_{turn-on}$[a] (V)	CIE_{1931}[b] [x, y]	EQE_{max}[c] [%]	CE_{max}[d] [cd A^{-1}]	PE_{max}[e] [lm W^{-1}]	L_{max}[f] [cd m^{-2}]	Device performance at 1000 cd m^{-2}	
							EQE [%]	PE [lm W^{-1}]
**1** (3%) in CBZ_2-F_1	3.4	(0.25, 0.42)	3.7	10.21	8.43	10307	2.5	3.87
**1** (5%) in CBZ_2-F_1	3.4	(0.25, 0.42)	3.8	10.69	9.07	10105	2.5	4.08
**1** (7%) in CBZ_2-F_1	3.4	(0.25, 0.42)	4.1	11.77	10.38	10876	2.7	4.31
**1** (9%) in CBZ_2-F_1	3.4	(0.24, 0.42)	3.9	11.13	9.72	9800	2.6	3.90
**1** (3%) in TBCPF	3.7	(0.24, 0.41)	3.6	9.28	7.47	4243	2.5	3.45
**1** (5%) in TBCPF	3.6	(0.24, 0.40)	3.5	9.39	7.72	4822	2.4	3.44
**1** (7%) in TBCPF	3.7	(0.25, 0.41)	5.3	14.54	12.34	4715	2.8	3.47
**1** (9%) in TBCPF	3.6	(0.24, 0.39)	3.9	10.19	8.88	4629	2.5	3.58
**2** (1%) in CBZ_2-F_1	3.5	(0.18, 0.15)	6.7	8.18	7.46	9124	4.4	2.64
**2** (2%) in CBZ_2-F_1	3.5	(0.18, 0.16)	8.0	9.97	8.95	8101	4.2	2.60
**2** (4%) in CBZ_2-F_1	3.5	(0.18, 0.16)	5.8	7.77	6.77	14143	4.1	2.54
**2** (6%) in CBZ_2-F_1	3.4	(0.18, 0.17)	6.0	7.77	6.46	14143	4.2	2.54
**2** (4%) in CBZ_2-F_1	4.2	(0.18, 0.15)	5.8	7.28	5.44	6378	4.1	2.50
**2** (6%) in TBCPF	4.2	(0.18, 0.16)	6.1	8.00	5.98	6125	4.0	2.59
**2** (8%) in TBCPF	4.3	(0.18, 0.16)	7.5	9.75	7.12	8763	4.4	2.82
**2** (10%) in TBCPF	4.3	(0.18, 0.15)	7.0	8.92	6.52	8942	4.4	2.76

[a]Turn on voltage at a brightness of 1 cd m^{-2}. [b]1 mA/cm^2. [c]External quantum efficiency. [d]Maximum current efficiency. [e]Maximum power efficiency. [f]Maximum luminance.

Other devices with **1** as the emitter:

Device **I**: ITO/MoO_3 (3 nm)/TAPC (50 nm)/**1** (7 wt%):TBCPF (10 nm)/TmPyPB (50 nm)/LiF (1 nm)/Al (100 nm)

Device **II**: ITO/MoO_3 (3 nm)/TAPC (50 nm)/**1** (7 wt%):TBCPF (15 nm)/TmPyPB (50 nm)/LiF (1 nm)/Al (100 nm)

Device **III**: ITO/MoO_3 (3 nm)/TAPC (60 nm)/**1** (7 wt%):TBCPF (20 nm)/TmPyPB (40 nm)/LiF (1 nm)/Al (100 nm)

Device **IV**: ITO/MoO_3 (3 nm)/TAPC (60 nm)/**1** (7 wt%):TBCPF (20 nm)/TmPyPB (50 nm)/LiF (1 nm)/Al (100 nm)

Device **V**: ITO/MoO_3 (3 nm)/TAPC (50 nm)/**1** (7 wt%):TBCPF (20 nm)/TmPyPB (60 nm)/LiF (1 nm)/Al (100 nm)

Table S3. Device performance with **1** as the emitter

Device	$V_{turn\text{-}on}$ [a] (V)	CIE_{1931} [b] [x, y]	EQE_{max} [c] [%]	CE_{max} [d] [cd A^{-1}]	PE_{max} [e] [lm W^{-1}]	L_{max} [f] [cd m^{-2}]	Device performance at 1000 cd m^{-2}	
							EQE [%]	PE [lm W^{-1}]
**I**	3.3	(0.25, 0.42)	2.8	5.09	6.93	4766	1.8	2.81
**II**	3.5	(0.24, 0.41)	3.6	5.87	8.48	4851	2.2	3.07
**III**	3.7	(0.23, 0.40)	2.7	4.26	5.98	3946	1.6	2.19
**IV**	3.6	(0.23, 0.40)	3.5	5.96	7.76	4711	2.3	3.02
**V**	3.7	(0.24, 0.41)	5.1	7.01	11.70	5024	2.6	3.34

[a]Turn on voltage at a brightness of 1 cd m^{-2}. [b]1 mA/cm^2. [c]External quantum efficiency. [d]Maximum current efficiency. [e]Maximum power efficiency. [f]Maximum luminance.

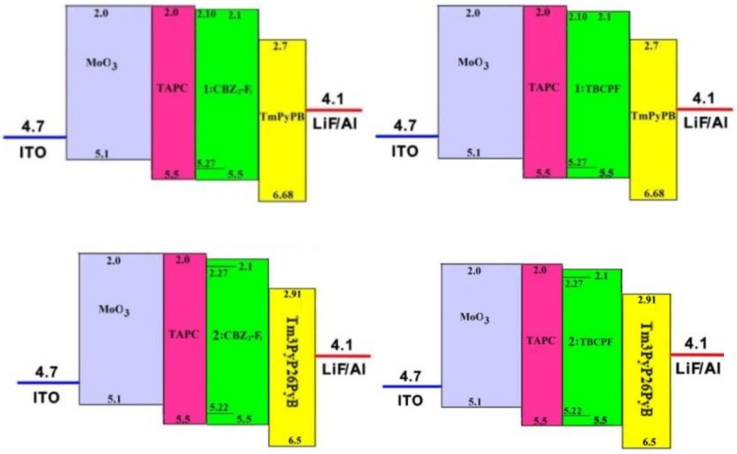

Fig. S6 Energy level diagrams (eV) of the fabricated devices (Device schematic).

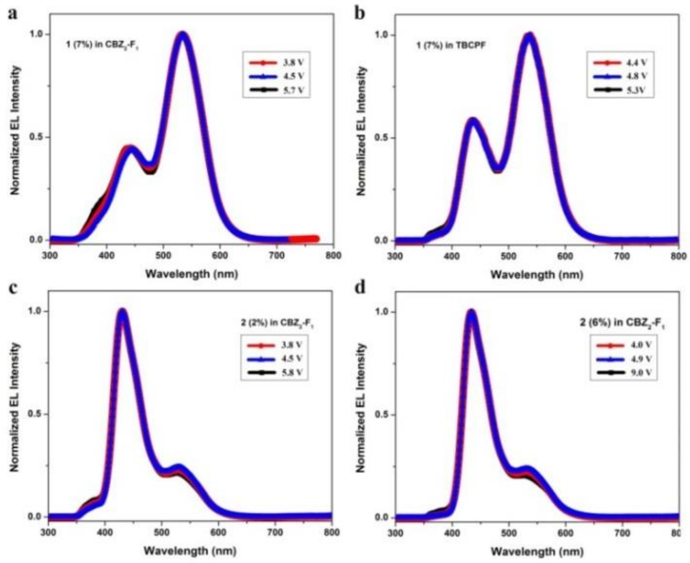

Fig. S7 EL spectra of devices **1** (7%) in CBZ$_2$-F$_1$ (a), **1** (7%) in TBCPF (b), **2** (2%) in CBZ$_2$-F$_1$ (c) and **2** (6%) in CBZ$_2$-F$_1$ (d) under different driving voltages.

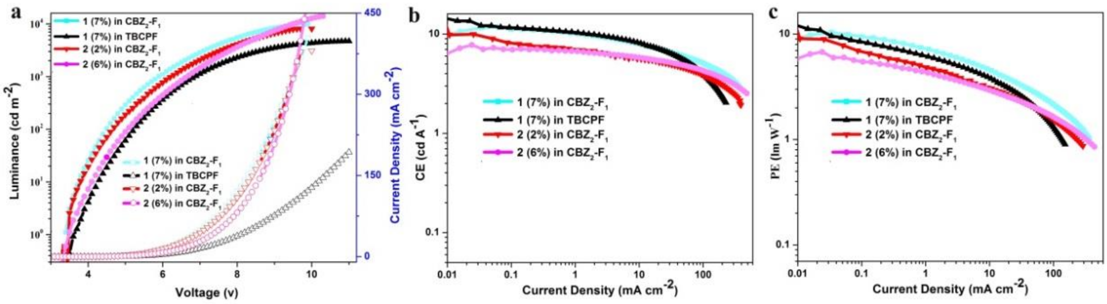

Fig. S8 (a) Brightness−current density−voltage (*B*−*J*−*V*) characteristics of devices **1** (7%) in CBZ$_2$-F$_1$, **1** (7%) in TBCPF, **2** (2%) in CBZ$_2$-F$_1$ and **2** (6%) in CBZ$_2$-F$_1$. (b) The EL efficiency-current density curves of devices **1** (7%) in CBZ$_2$-F$_1$, **1** (7%) in TBCPF, **2** (2%) in CBZ$_2$-F$_1$ and **2** (6%) in CBZ$_2$-F$_1$. (c) The PE efficiency-current density curves of devices **1** (7%) in CBZ$_2$-F$_1$, **1** (7%) in TBCPF, **2** (2%) in CBZ$_2$-F$_1$ and **2** (6%) in CBZ$_2$-F$_1$.

VII. Absorption spectra of 1 and 2 in film

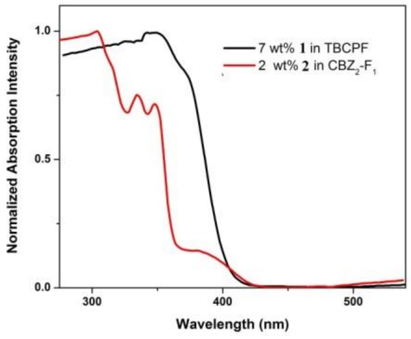

Fig. S9 Normalized absorption spectra of **1** in film (7 wt% in TBCPF) and **2** in film (2 wt% in CBZ$_2$-F$_1$).

VIII. Transient emission spectra of 1, 2 and DHPZ-2BN and oxygen quenching experiments.

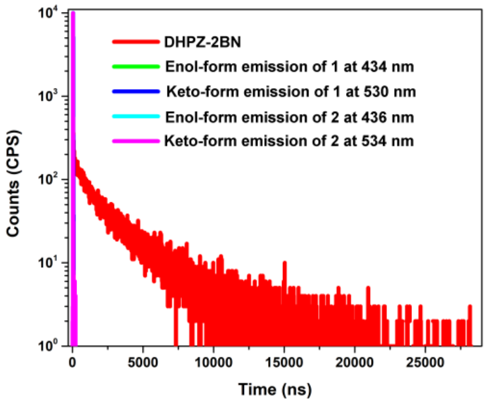

Fig. S10 Transient emission spectra of **1**, **2** and DHPZ-2BN in doped film at room temperature. 5 wt% DHPZ-2BN in TBCPF; enol-form emission of **1** at 434 nm and keto-form emission of **1** at 530 nm in doped film (7 wt% **1** in TBCPF); enol-form emission of **2** at 436 nm and keto-form emission of **2** at 534 nm in doped film (2 wt% **2** in CBZ_2-F_1).

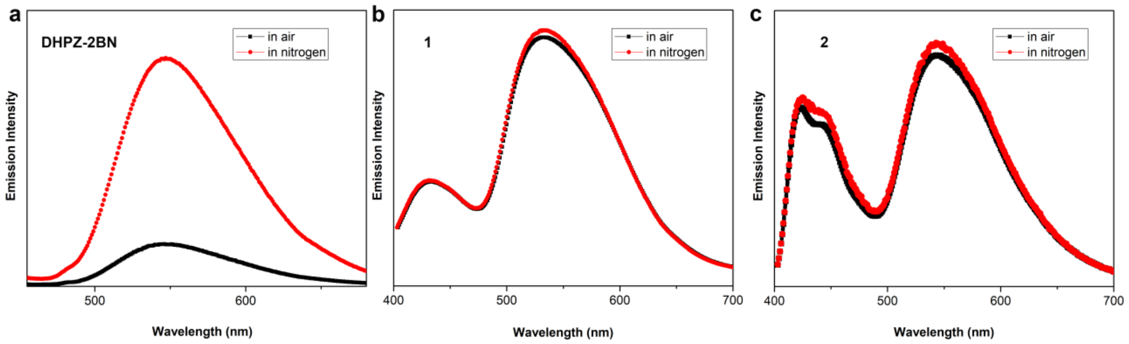

Fig. S11 (a) PL spectra of DHPZ-2BN in toluene (concentration: 1 × 10^{-5} mol L^{-1}) in air and under an nitrogen atmosphere at room teperature. (b) PL spectra of **1** in toluene (concentration: 5 × 10^{-5} mol L^{-1}) in air and under an nitrogen atmosphere at room teperature. (c) PL spectra of **2** in toluene (concentration: 5 × 10^{-5} mol L^{-1}) in air and under an nitrogen atmosphere at room teperature. Nitrogen and air bubbling for 30 min.

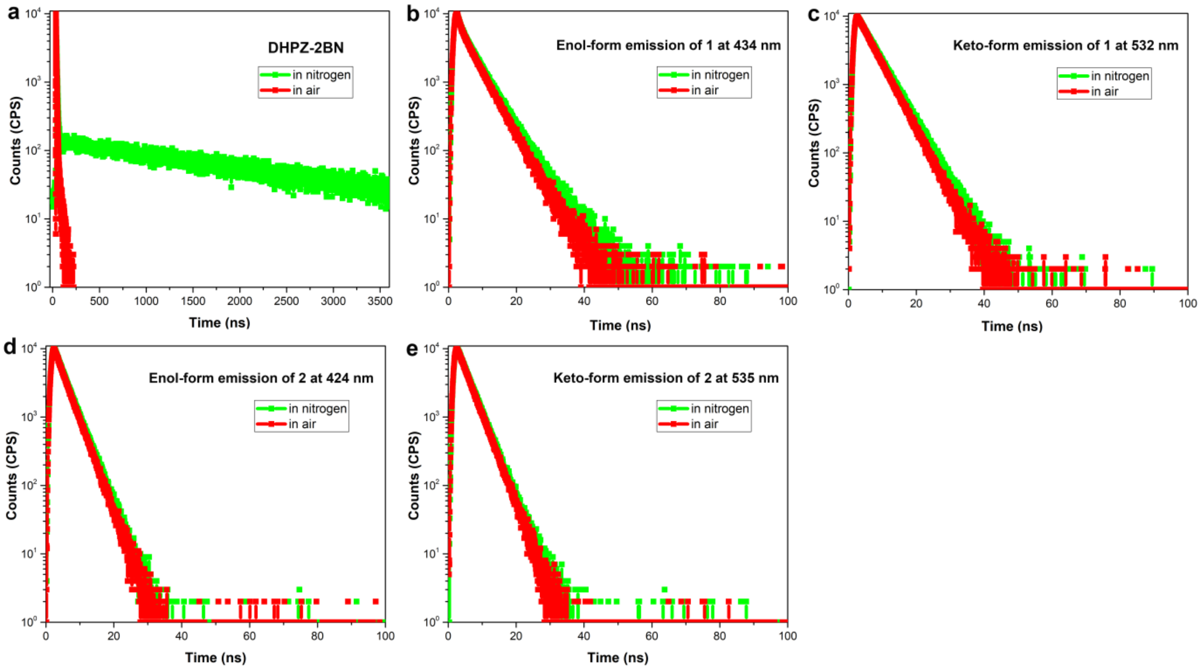

Fig. S12 (a) Transient emission spectra of DHPZ-2BN in toluene (1×10^{-5} mol L^{-1}) at room temperature under different atmospheres. (b), (c)Transient emission spectra of **1** in toluene (5×10^{-5} mol L^{-1}) at room temperature under different atmospheres. (d), (e) Transient emission spectra of **2** in toluene (5×10^{-5} mol L^{-1}) at room temperature under different atmospheres. Nitrogen and air bubbling for 30 min.

Table S4. Excited state lifetimes of DHPZ-2BN (1×10^{-5} mol L^{-1}), **1** (5×10^{-5} mol L^{-1}) and **2** (5×10^{-5} mol L^{-1}) in toluene in air and under an nitrogen atmosphere at room teperature.[a]

	In nitrogen		In air	
	Life time	Average life time	Life time	Average life time
DHPZ-2BN	τ_1 = 6.79 ns, (25%) τ_2 = 1891 ns, (75%) χ^2 =1.02	1419.94 ns	τ_1 = 4.92 ns, (94%) τ_2 = 25.8 ns, (6%) χ^2 =1.03	6.17 ns
Enol-form emission of **1** at 434 nm	τ_1 = 0.93 ns, (54%) τ_2 = 5.01 ns, (46%) χ^2 =1.03	2.83 ns	τ_1 = 0.81 ns, (55%) τ_2 = 4.69 ns, (45%) χ^2 =1.02	2.56 ns
Keto-form emission of **1** at 532 nm	τ_1 = 4.74 ns, χ^2 = 1.08	4.74 ns	τ_1 = 4.54 ns, χ^2 = 1.06	4.54 ns
Enol-form emission of **2** at 424 nm	τ_1 = 0.62 ns, (5%) τ_2 = 3.41 ns, (95%) χ^2 =1.00	3.27 ns	τ_1 = 0.49 ns, (5%) τ_2 = 3.21 ns, (95%) χ^2 =1.10	3.07 ns

Keto-form emission of **2** at 535 nm	τ$_1$ = 3.45 ns, χ^2 =1.00	3.45 ns	τ$_1$ = 3.33 ns, χ^2 =1.02	3.33 ns

[a]The excited state lifetimes were determined on a HORIBA TEMPRO-01 instrument.

IX. Excited state lifetimes of 1 and 2 in other solvents

Table S5. Excited state lifetimes of **1** (5×10^{-5} mol L^{-1}) in different solvents at room temperature[a]

Solvent	Enol-form		Keto-form	
	Life time	Average life time	Life time	Average life time
cyclohexane	τ_1 = 0.89 ns, (34%)<br>τ_2 = 3.53 ns, (66%)<br>χ^2 =1.08	2.69 ns	τ_1 = 3.55 ns,<br>χ^2 = 1.06	3.55 ns
benzene	τ_1 = 1.02 ns, (54%)<br>τ_2 = 5.00 ns, (46%)<br>χ^2 =1.05	2.87 ns	τ_1 = 4.62 ns,<br>χ^2 = 1.05	4.62 ns
dioxane	τ_1 = 1.22 ns, (82%)<br>τ_2 = 6.35 ns, (18%)<br>χ^2 = 1.18	2.16 ns	τ_1 = 4.39 ns,<br>χ^2 = 1.06	4.39 ns
butyl ether	τ_1 = 1.18 ns, (75%)<br>τ_2 = 4.76 ns, (25%)<br>χ^2 = 1.07	2.07 ns	τ_1 = 4.42 ns,<br>χ^2 = 1.03	4.42 ns
chlorobenzene	τ_1 = 2.50 ns, (39%)<br>τ_2 = 5.68 ns, (61%)<br>χ^2 = 1.13	4.45 ns	τ_1 = 4.98 ns,<br>χ^2 = 1.08	4.98 ns
chloroform	τ_1 = 2.05 ns, (78%)<br>τ_2 = 8.21 ns, (22%)<br>χ^2 = 1.01	3.41 ns	τ_1 = 4.77 ns,<br>χ^2 = 1.06	4.77 ns
ethyl acetate	τ_1 = 2.91 ns, (77%)<br>τ_2 = 6.83 ns, (23%)<br>χ^2 = 1.17	3.80 ns	τ_1 = 4.51 ns,<br>χ^2 = 1.12	4.51 ns
tetrahydrofuran	τ_1 = 3.72 ns, (89%)<br>τ_2 = 9.35 ns, (11%)<br>χ^2 =1.17	4.35 ns	τ_1 = 4.64 ns,<br>χ^2 = 1.09	4.64 ns
methylene chloride	τ_1 = 2.02 ns, (92%)<br>τ_2 = 6.14 ns, (8%)<br>χ^2 = 1.10	2.36 ns	τ_1 = 3.78 ns, (69%)<br>τ_2 = 6.70 ns, (31%)<br>χ^2 = 1.12	4.69 ns
1,2-dichloroethane	τ_1 = 2.55 ns, (71%)<br>τ_2 = 7.19 ns, (29%)<br>χ^2 = 1.08	3.88 ns	τ_1 = 5.05 ns, (88%)<br>τ_2 = 9.21 ns, (12%)<br>χ^2 = 1.02	5.55 ns

[a]The excited state lifetimes were determined on a HORIBA TEMPRO-01 instrument.

Table S6. Excited state lifetimes of **2** (5×10^{-5} mol L^{-1}) in different solvents at room temperature.[a]

Solvent	Enol-form		Keto-form	
	Life time	Average life time	Life time	Average life time

cyclohexane	τ_1 = 2.67 ns, χ^2 = 1.15	2.67 ns	τ_1 = 2.81 ns, χ^2 = 1.02	2.81 ns
hexane	τ_1 = 2.92 ns, χ^2 = 1.06	2.92 ns	τ_1 = 3.09 ns, χ^2 = 1.07	3.09 ns
benzene	τ_1 = 2.99 ns, χ^2 = 1.25	2.99 ns	τ_1 = 3.18 ns, χ^2 = 1.09	3.18 ns
dioxane	τ_1 = 0.13 ns, (89%) τ_2 = 2.75 ns, (11%) χ^2 = 1.14	0.41 ns	τ_1 = 2.95 ns, χ^2 = 1.06	2.95 ns
butyl ether	τ_1 = 1.02 ns, (51%) τ_2 = 2.85 ns, (41%) χ^2 = 1.12	1.92 ns	τ_1 = 2.98 ns, χ^2 = 1.01	2.98 ns
chlorobenzene	τ_1 = 2.79 ns, χ^2 = 1.09	2.79 ns	τ_1 = 3.09 ns, χ^2 = 1.07	3.09 ns
chloroform	τ_1 = 2.89 ns, χ^2 = 1.14	2.89 ns	τ_1 = 3.14 ns, χ^2 = 1.04	3.14 ns
ethyl acetate	τ_1 = 2.72 ns, χ^2 = 1.17	2.72 ns	τ_1 = 2.88 ns, χ^2 = 0.97	2.88 ns
tetrahydrofuran	τ_1 = 2.75 ns, χ^2 = 1.15	2.75 ns	τ_1 = 2.92 ns, χ^2 = 1.03	2.92 ns
methylene chloride	τ_1 = 2.86 ns, χ^2 = 1.13	2.86 ns	τ_1 = 2.93 ns, χ^2 = 1.02	2.93 ns
1,2-dichloroethane	τ_1 = 2.78 ns, χ^2 = 1.06	2.78 ns	τ_1 = 2.86 ns, χ^2 = 1.09	2.86 ns
acetonitrile	τ_1 = 3.13 ns, χ^2 = 1.05	3.13 ns	τ_1 = 3.16 ns, χ^2 = 1.08	3.16 ns

[a]The excited state lifetimes were determined on a HORIBA TEMPRO-01 instrument.

X. Excited state lifetimes of 1 and 2 in film

Table S7. Excited state lifetimes of **1** in doped film (7 wt% in TBCPF)[a]

Temperature（K）	Enol-form		Keto-form	
	Life time	Average life time	Life time	Average life time
77	τ_1 = 0.32 ns, (74%) τ_2 = 4.51 ns, (26%) χ^2 = 1.04	1.41 ns	τ_1 = 2.42 ns, (33%) τ_2 = 5.08 ns, (67%) χ^2 = 1.17	4.20 ns
107	τ_1 = 1.43 ns, (45%) τ_2 = 4.85 ns, (55%) χ^2 = 1.02	3.32 ns	τ_1 = 2.86ns, (38%) τ_2 = 5.36 ns, (62%) χ^2 = 1.04	4.40 ns
137	τ_1 = 1.42 ns, (44%) τ_2 = 4.89 ns, (56%) χ^2 =1.03	3.38 ns	τ_1 = 2.85ns, (38%) τ_2 = 5.38 ns, (62%) χ^2 = 1.02	4.43 ns
167	τ_1 = 1.52 ns, (44%) τ_2 = 4.93 ns, (56%) χ^2 = 1.05	3.42 ns	τ_1 = 2.93ns, (39%) τ_2 = 5.41 ns, (61%) χ^2 = 1.19	4.45 ns
197	τ_1 = 1.47 ns, (43%) τ_2 = 4.89 ns, (57%) χ^2 = 1.12	3.41 ns	τ_1 = 3.03 ns, (42%) τ_2 = 5.46 ns, (58%) χ^2 = 1.08	4.45 ns
227	τ_1 = 1.43 ns, (43%) τ_2 = 4.87 ns, (57%) χ^2 = 1.11	3.39 ns	τ_1 = 2.79 ns, (36%) τ_2 = 5.28 ns, (64%) χ^2 = 1.11	4.40 ns

257	τ_1 = 1.39 ns, (42%)<br>τ_2 = 4.78 ns, (58%)<br>χ^2 = 1.20	3.36 ns	τ_1 = 2.87 ns, (40%)<br>τ_2 = 5.35 ns, (60%)<br>χ^2 = 1.08	4.37 ns
287	τ_1 = 1.40 ns, (42%)<br>τ_2 = 4.78 ns, (58%)<br>χ^2 = 1.17	3.35 ns	τ_1 = 2.68 ns, (38%)<br>τ_2 =5.20 ns, (62%)<br>χ^2 = 1.01	4.24 ns
317	τ_1 = 1.22 ns, (43%)<br>τ_2 = 4.57 ns, (57%)<br>χ^2 = 1.21	3.13 ns	τ_1 = 2.67 ns, (40%)<br>τ_2 = 5.16 ns, (60%)<br>χ^2 = 1.10	4.17 ns

[a]The excited state lifetimes were determined on a HORIBA TEMPRO-01 instrument.

Table S8. Excited state lifetimes of **2** in doped film (2 wt% in CBZ_2-F_1)[a]

Temperature（K）	Enol-form		Keto-form	
	Life time	Average life time	Life time	Average life time
77	τ_1 = 1.12 ns, (79%)<br>τ_2 = 3.02 ns, (21%)<br>χ^2 = 1.16	1.53 ns	τ_1 = 2.00 ns, (61%)<br>τ_2 = 4.49 ns, (39%)<br>χ^2 = 1.01	2.96 ns
107	τ_1 = 1.06 ns, (80%)<br>τ_2 = 3.05 ns, (20%)<br>χ^2 = 1.19	1.45 ns	τ_1 = 1.86 ns, (60%)<br>τ_2 = 4.48 ns, (40%)<br>χ^2 = 1.17	2.91 ns
137	τ_1 = 1.06 ns, (81%)<br>τ_2 = 3.11 ns, (19%)<br>χ^2 = 0.98	1.44 ns	τ_1 = 1.84 ns, (59%)<br>τ_2 = 4.50 ns, (41%)<br>χ^2 = 1.26	2.92 ns
167	τ_1 = 1.05 ns, (81%)<br>τ_2 = 3.05 ns, (19%)<br>χ^2 = 1.00	1.43 ns	τ_1 = 1.91 ns, (60%)<br>τ_2 = 4.53 ns, (40%)<br>χ^2 = 1.17	2.95 ns
197	τ_1 = 1.02 ns, (80%)<br>τ_2 = 3.00 ns, (20%)<br>χ^2 = 1.17	1.41 ns	τ_1 = 1.93 ns, (62%)<br>τ_2 = 4.56 ns, (38%)<br>χ^2 = 1.05	2.94 ns
227	τ_1 = 1.08 ns, (80%)<br>τ_2 = 3.05 ns, (20%)<br>χ^2 = 1.02	1.47 ns	τ_1 = 1.90 ns, (62%)<br>τ_2 = 4.51 ns, (38%)<br>χ^2 = 1.06	2.90 ns
257	τ_1 = 1.06 ns, (80%)<br>τ_2 = 3.04 ns, (20%)<br>χ^2 = 1.03	1.46 ns	τ_1 = 1.88 ns, (62%)<br>τ_2 = 4.41 ns, (38%)<br>χ^2 = 1.05	2.85 ns
287	τ_1 = 1.08 ns, (79%)<br>τ_2 = 3.01 ns, (21%)<br>χ^2 = 1.04	1.49 ns	τ_1 = 1.82 ns, (62%)<br>τ_2 = 4.35 ns, (38%)<br>χ^2 = 1.16	2.78 ns
300	τ_1 = 1.05 ns, (77%)<br>τ_2 = 2.93 ns, (23%)<br>χ^2 = 1.10	1.48 ns	τ_1 = 1.80 ns, (62%)<br>τ_2 = 4.21 ns, (38%)<br>χ^2 = 1.04	2.72 ns

[a]The excited state lifetimes were determined on a HORIBA TEMPRO-01 instrument.

XI. Solvatochromic effect of 1 and 2

Solvent-dependent spectral shifts are often interpreted in terms of the Lippert−Mataga equation (S1a),[4,5] which describes the solvatochromic Stokes shift $\Delta\nu$ (between the maxima

of absorption and fluorescence emission, and expressed in wavenumbers) as a function of the dipole moment change ($\Delta\mu = \mu_e - \mu_g$) of the dye upon excitation:

$$\Delta\nu = \nu_{abs} - \nu_{em} = \frac{2\Delta f}{hca^3}(\mu_e - \mu_g)^2 + const \qquad \text{(S1a)}$$

In eq. S1a, h is Planck's constant, c is the velocity of light, and a is the Onsager cavity radius. The Onsager cavity radius (a) is estimated from quantumchemical calculations by using density functional theory method under the B3LYP/6-31g* level.[5,6] The Onsager radius **1** and **2** are calculated to be 4.48 Å and 6.03 Å as the effective radius. μ_g and μ_e denote the dipole moments of the dye in ground and excited states, respectively. The μ_g can be estimated at the level of wB97X/6-311+g(d, p) level with the Gaussian 09 package.[7] The orientational polarizability of solvent (Δf) can be calculated from the dielectric constants ε and the refractive indices n of the solvent using the following equation (S1b).

$$\Delta f = \frac{\varepsilon - 1}{2\varepsilon + 1} - \frac{n^2 - 1}{2n^2 + 1} \qquad \text{(S1b)}$$

The dipole moment change ($\Delta\mu$) was obtained from the solvatochromic shift in various solvents based on eq. S1a and S1b. The enol-form of **1** and **2** displayed a good linear correlation between Stokes shift (ν_a - ν_f) and solvent polarity manifests (f), which show only one slope value of 7008 (r = 0.99) and 10577 (r = 0.98), respectively. The dipole moment change of **1** and **2** between the enol-form excited state and ground state ($\Delta\mu = \mu_e - \mu_g$), which can serve as an empirical scale to evaluate the strength of the intramolecular charge transfer (ICT),[5] was calculated to be 8.0 D and 15.2 D and the corresponding μ_e were thus estimated to be 11.9 D and 18.9 D, respectively.

Table S9. Photophysical properties of **1** in different solvents[a]

Solvent	**Δ*f***	**1**					Φ_f(%)
		λ_{abs}	$\lambda_{em\text{-}enol}$	ν_a - ν_f	$\lambda_{em\text{-}keto}$	ν_a - ν_f	

		(nm)	(nm)	(cm^{-1})	(nm)	(cm^{-1})	
cyclohexane	0.0033	319	429	8038	526	12336	14.1
benzene	0.0026	321	433	8058	533	12391	24.2
toluene	0.014	321	434	8111	532	12356	26.3
dioxane	0.021	320	433	8155	533	12488	22.7
butyl ether	0.096	316	434	8604	530	12778	22.3
chlorobenzene	0.143	321	453	9078	535	12461	22.5
chloroform	0.149	319	450	9126	533	12586	23.7
ethyl acetate	0.200	319	455	9369	533	12586	19.9
tetrahydrofuran	0.210	321	460	9414	535	12461	18.2
methylene chloride	0.217	321	463	9558	530	12285	27.4
1,2-dichloroethane	0.222	320	464	9698	534	12523	18.7

$^{a}\lambda_{abs}$ = absorption maximum, $\lambda_{em\text{-}enol}$ = enol-form emission, $\lambda_{em\text{-}keto}$ = keto-form emission, and Φ_f = fluorescence absolute quantum yield.

Table S10. Photophysical properties of **2** in different solvents^{a}

Solvent	**Δ*f***	**2**					Φ_f (%)
		λ_{abs} (nm)	$\lambda_{em\text{-}enol}$ (nm)	$\nu_a - \nu_f$ (cm^{-1})	$\lambda_{em\text{-}keto}$ (nm)	$\nu_a - \nu_f$ (cm^{-1})	
cyclohexane	0.0033	369	412	2828	538	8513	47.6
hexane	0.0012	371	413	2741	541	8470	47.1
benzene	0.0026	370	421	3274	547	8745	50.9
dioxane	0.021	372	426	3408	548	8634	41.5
butyl ether	0.096	371	429	3664	546	8639	37.6
chlorobenzene	0.143	372	442	4257	546	8567	64.1
chloroform	0.149	373	444	4287	547	8528	41.0
ethyl acetate	0.200	371	452	4830	547	8673	37.1
tetrahydrofuran	0.210	369	457	5218	547	8819	44.5
methylene chloride	0.217	372	465	5376	545	8533	52.3
1,2-dichloroethane	0.222	371	468	5586	543	8538	51.9
acetonitrile	0.305	372	480	6048	540	8363	40.2

$^{a}\lambda_{abs}$ = absorption maximum, $\lambda_{em\text{-}enol}$ = enol-form emission, $\lambda_{em\text{-}keto}$ = keto-form emission, and Φ_f = fluorescence absolute quantum yield.

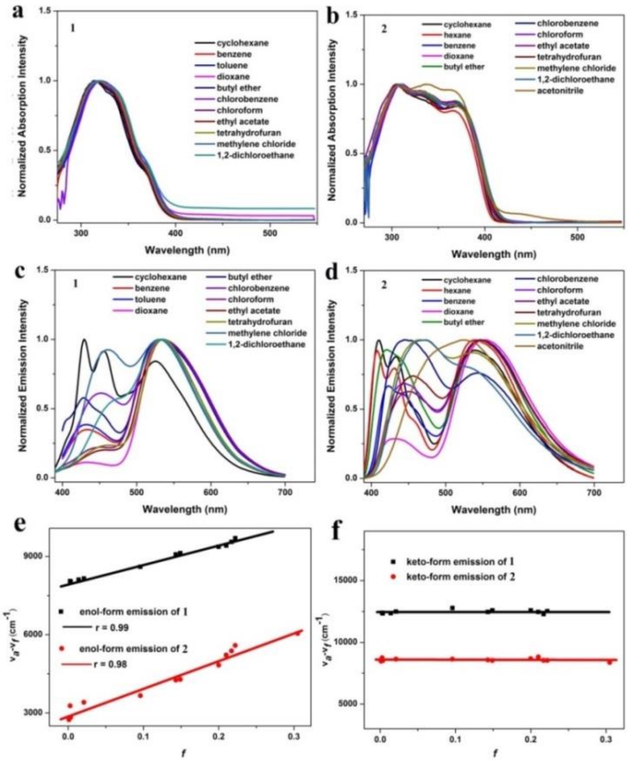

Fig. S13 (a) Normalized absorption spectra of **1** in different solvents (concentration: 5×10^{-5} mol L^{-1}). (b) Normalized absorption spectra of **2** in different solvents (concentration: 5×10^{-5} mol L^{-1}). (c) Normalized emission spectra of **1** in different solvents (concentration: 5×10^{-5} mol L^{-1}). (d) Normalized emission spectra of **2** in different solvents (concentration: 5×10^{-5} mol L^{-1}). (e) and (f) Linear fitting of Lippert–Mataga model (*f*: orientation polarization of solvent media; $\nu_a - \nu_f$: Stokes shift of enol-form and keto-form of **1** and **2** in different solvent).

XII. Calculated energy landscape and natural transition orbitals (NTOs) of the singlet and triplet states for 1 and 2

The ground-state geometries were optimized under the B3LYP/6-311+G(d, p) level. Based on the optimized ground state configuration, the high excitation energy levels of singlet and triplet states were evaluated using the TD-M06-2X/6-311+G(d, p) method. In order to examine the character of excited-states, natural transition orbitals (NTOs) were evaluated for the first five excited-states, involving both singlet and triplet states. This approach provides the most compact representation of the electronic transitions in terms of an expansion into single particle orbitals by diagonalizing the transition density matrix associated with each excitation. All calculations were performed using the Gaussian 09 program package.[7]

Table S11. Energy level and oscillator strengths of **1**

Excited States	S energy level (eV)	T energy level (eV)	S Oscillator Strength
1	3.707	2.825	1.112
2	3.995	3.237	0.734
3	4.081	3.415	0.146
4	4.156	3.545	0.343
5	4.276	3.715	0.302
6	4.356	3.767	0.038
7	4.407	3.976	0.027
8	4.630	4.154	0.004
9	4.658	4.233	0.052
10	4.671	4.318	0.002

Table S12. Energy level and oscillator strengths of **2**

Excited States	S energy level (eV)	T energy level (eV)	S Oscillator Strength
1	3.544	2.769	1.769
2	3.927	3.168	0.054
3	4.000	3.300	0.836
4	4.087	3.512	0.050
5	4.096	3.539	0.030
6	4.162	3.568	0.152
7	4.257	3.718	0.210
8	4.278	3.736	0.338
9	4.294	3.775	0.008
10	4.328	3.999	0.010

Excited States	Hole		Particle
S_1	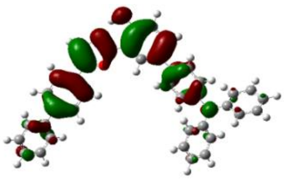	⇒ 91.6%	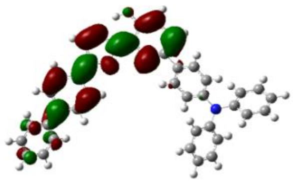

S_2	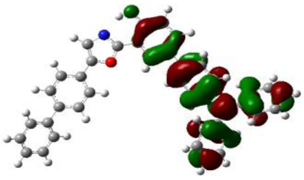	94.9%	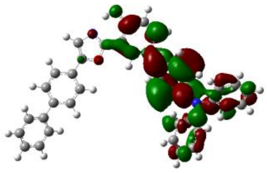
S_3	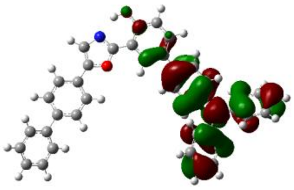	86.9%	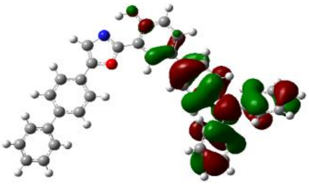
S_4	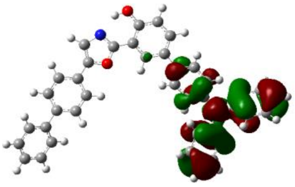	78.9%	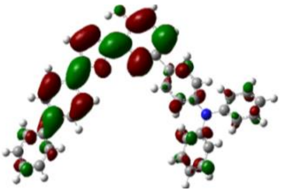
S_5	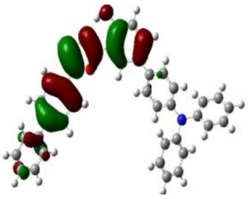	98.2%	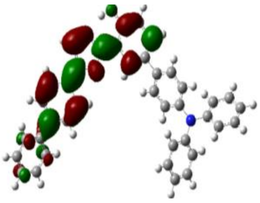

Fig. S14 Natural transition orbitals of singlet excited states (S_1 to S_5) of **1**.

Excited States	Hole		Particle
T_1	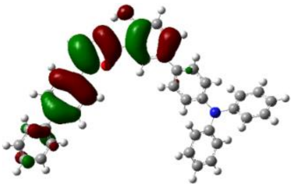	87.3%	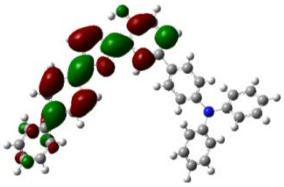
T_2	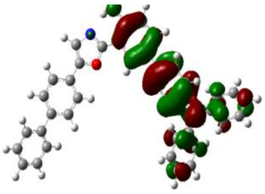	78.1%	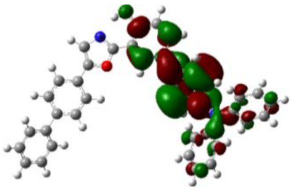
T_3	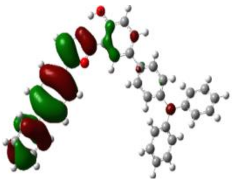	24.9%	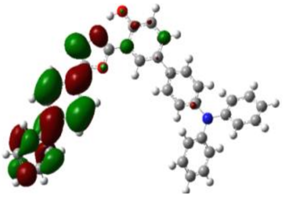

	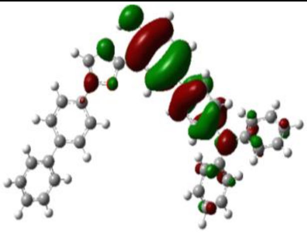	62.1%	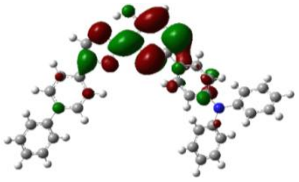
T_4	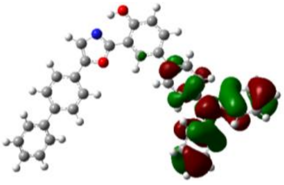	85.2%	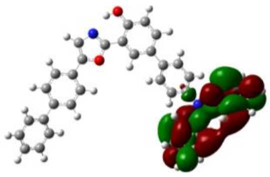
T_5	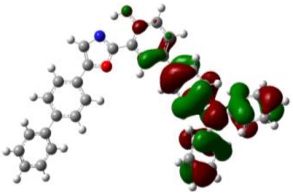	81.2%	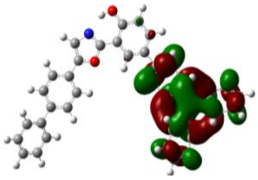

Fig. S15 Natural transition orbitals of triplet excited states (T_1 to T_5) of **1**.

Excited States	Hole	%	Particle
S_1	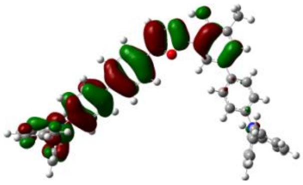	86.1%	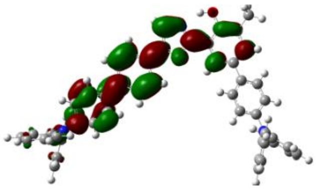
S_2	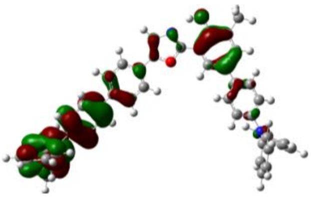	25.2%	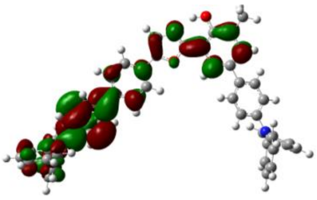
	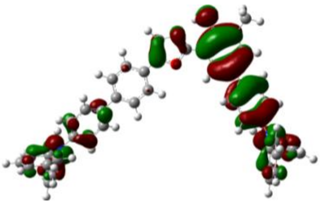	71.2%	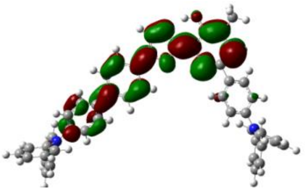
S_3	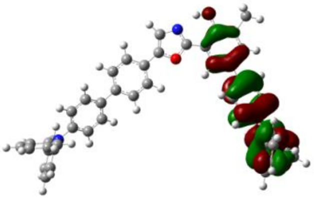	95.1%	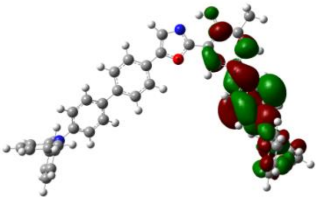

S_4	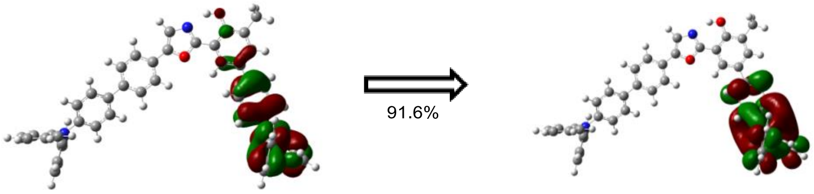
S_5	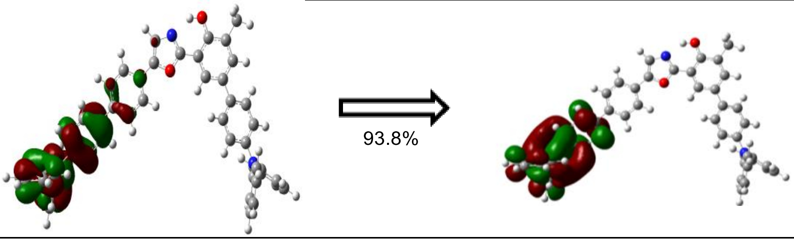

Fig. S16 Natural transition orbitals of singlet excited states (S_1 to S_5) of **2**.

Excited States	Hole → Particle
T_1	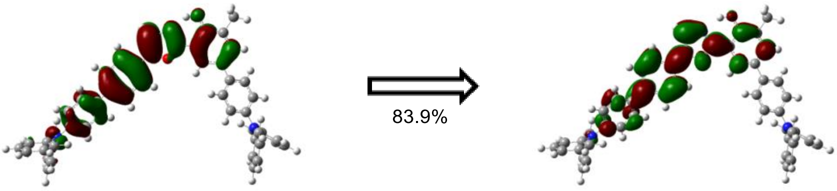
T_2	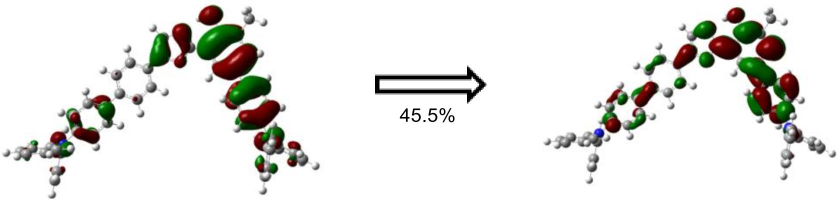
	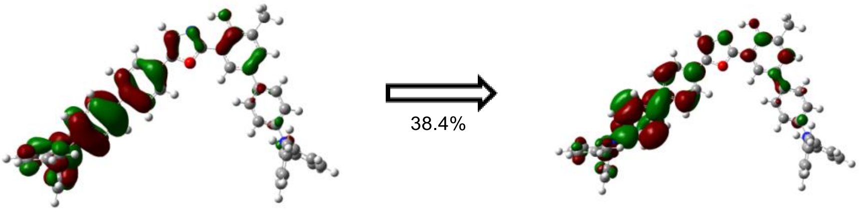
	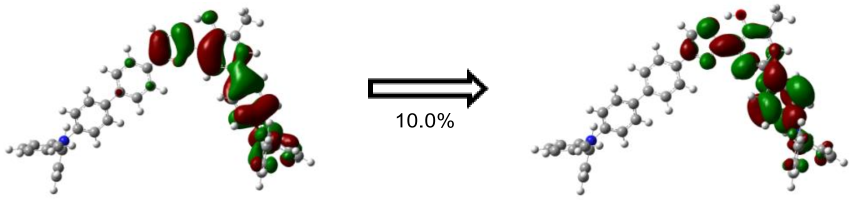
T_3	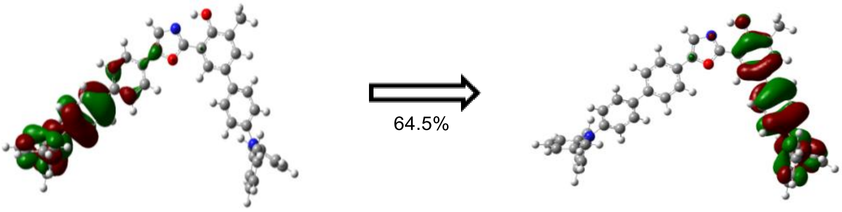

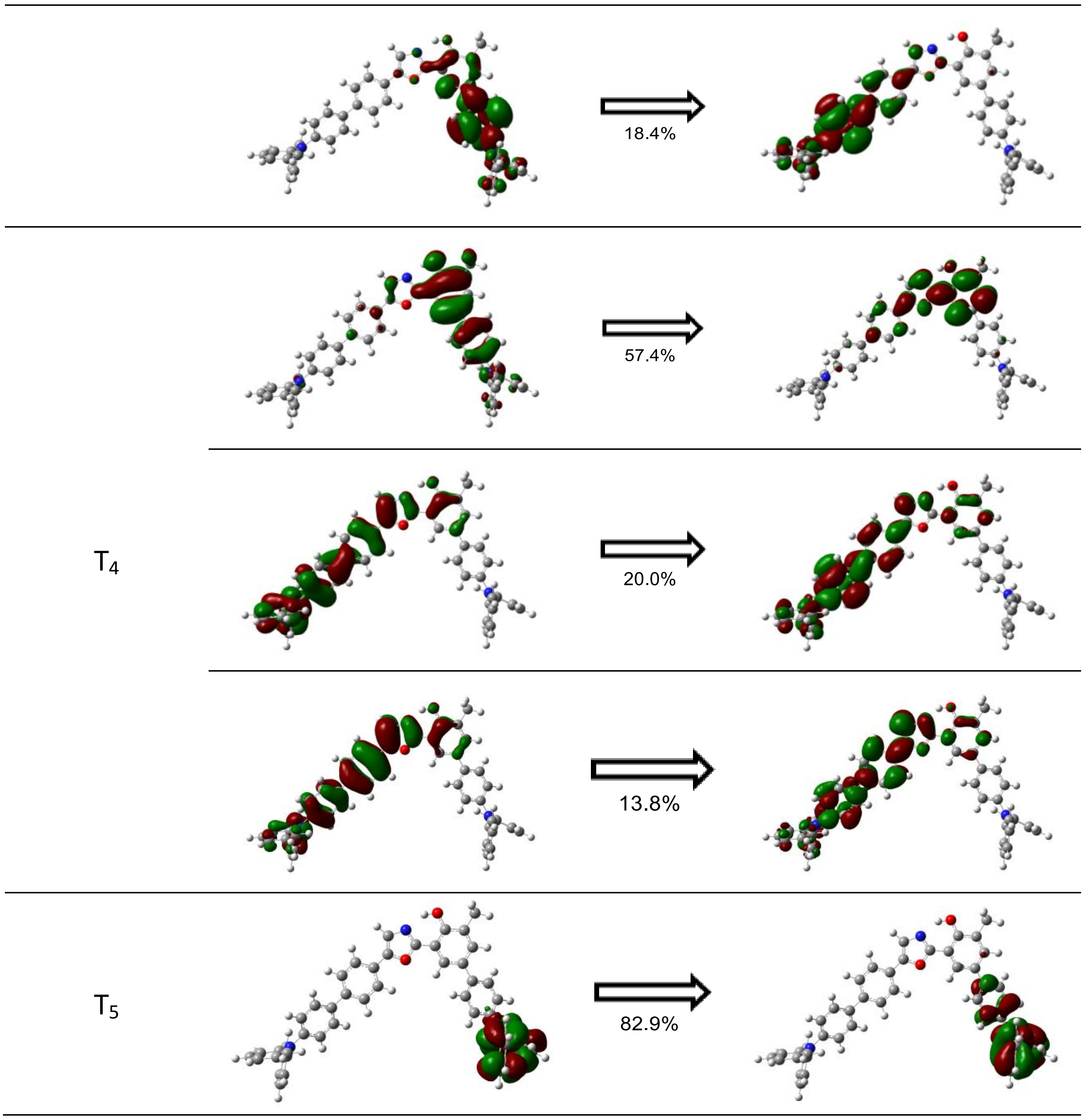

Fig. S17 Natural transition orbitals of triplet excited states (T_1 to T_5) of **2**.

XIII. References

1 (*a*) Y. Tamura, J. Minamikawa, K. Sumoto, S. Fujii and M. Ikeda, *J. Org. Chem.* 1973, **38**, 1239; (*b*) C. R. Johnson, R. A. Kirchhoff and H. G. Corkins, *J. Org. Chem.* 1974, **39**, 2458; (*c*) W. A. Wasylenko, N. Kebede, B. M. Showalter, N. Matsunaga, A. P. Miceli, Y. Liu, L. R. Ryzhkov, C. M. Hadad and J. P. Toscano, *J. Am. Chem. Soc.* 2006, **128**, 13142; (*d*) G. J. L. Bernardes, J. M. Chalker, J. C. Errey and B. G. Davis, *J. Am. Chem. Soc.* 2008, **130**, 5052.

2 J. Lee, K. Shizu, H. Tanaka, H. Nakanotani, T. Yasuda, H. Kaji and C. Adachi, *J. Mater. Chem. C*, 2015, **3**, 2175.

3 (*a*) Y. Endo, K. Shudo and T. Okamoto, *Synthesis* 1980, 461; (*b*) B. Li, J. Lan, D. Wu and J. You, *Angew. Chem. Int. Ed.* 2015, **54**, 14008.

4 (*a*) N. Mataga, Y. Kaifu and M. Koizumi, *Bull. Chem. Soc. Jpn*. 1956, **29**, 465; (*b*) V. E. Lippert, *Elektrochem.* 1957, **61**, 962.

5 M. Jia, X. Ma, L. Yan, H. Wang, Q. Guo, X. Wang, Y. Wang, X. Zhan and A. Xia, *J. Phys. Chem. A.*, 2010, **114**, 7345.

6 (*a*) J. Karpiuk, *J. Phys. Chem. A* 2004, **108**, 11183; (*b*) Y. Gong, X. Guo, S. Wang, H. Su, A. Xia, Q. He and F. Bai, *J. Phys. Chem. A* 2007, **111**, 5806.

7 M. J. Frisch, G. W. Trucks, H. B. Schlegel, G. E. Scuseria, M. A. Robb , J. R. Cheeseman, G. Scalmani, V. Barone, B. Mennucci , G. A. Petersson, H. Nakatsuji, M. Caricato, X. Li, H. P. Hratchian, A. F. Izmaylov, J. Bloino, G. Zheng, J. L. Sonnenberg, M. Hada, M. Ehara, K. Toyota, R. Fukuda, J. Hasegawa, M. Ishida, T. Nakajima, Y. Honda, O. Kitao, H. Nakai, T. Vreven, J. A. Montgomery, Jr., J. E. Peralta, F. Ogliaro, M. Bearpark, J. J. Heyd, E. Brothers, K. N. Kudin, V. N. Staroverov, R. Kobayashi, J. Normand, K. Raghavachari, A. Rendell, J. C. Burant, S. S. Iyengar, J. Tomasi, M. Cossi, N. Rega, J. M. Millam, M. Klene, J. E. Knox, J. B. Cross, V. Bakken, C. Adamo, J. Jaramillo, R. Gomperts, R. E. Stratmann, O. Yazyev, A. J. Austin, R. Cammi, C. Pomelli, J. W. Ochterski, R. L. Martin, K. Morokuma, V. G. Zakrzewski, G. A. Voth, P. Salvador, J. J. Dannenberg, S. Dapprich, A. D. Daniels, O. Farkas, J. B. Foresman, J. V. Ortiz, J. Cioslowski and A. D. J. Fox, Gaussian 09, Revision A.02 , Gaussian Inc., Wallingford, CT, 2009.

XIV. Copies of ^{1}H and ^{13}C NMR spectra

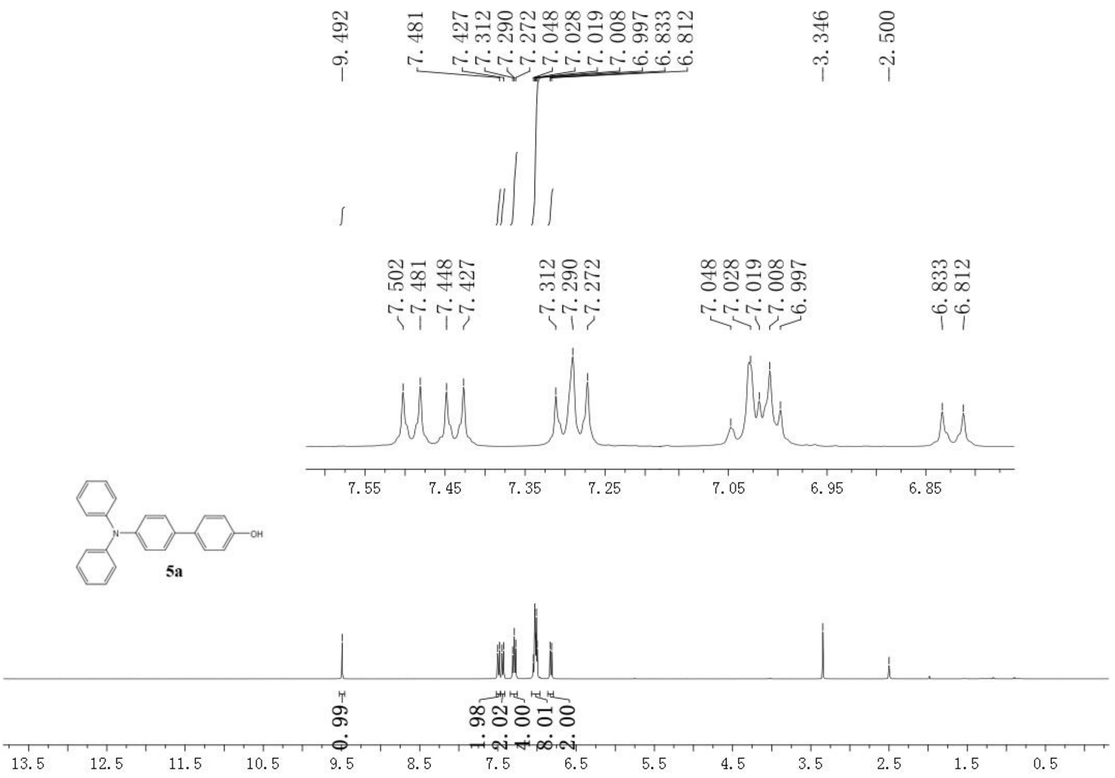

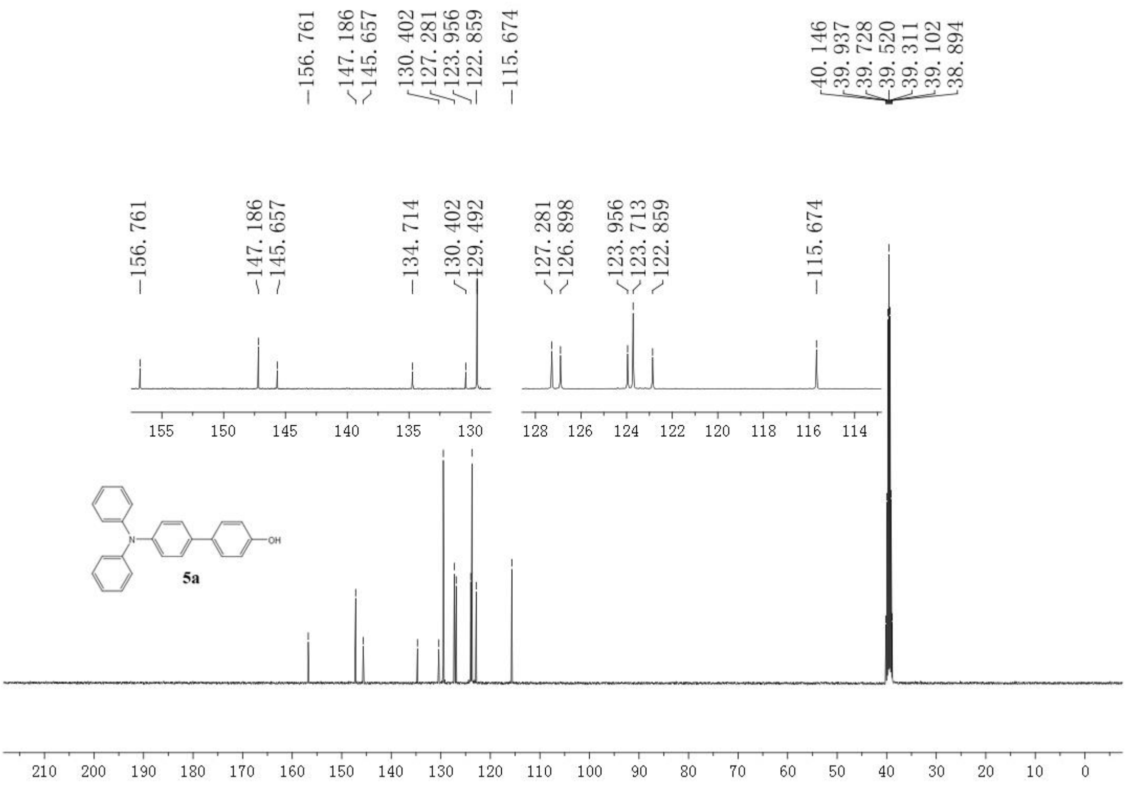

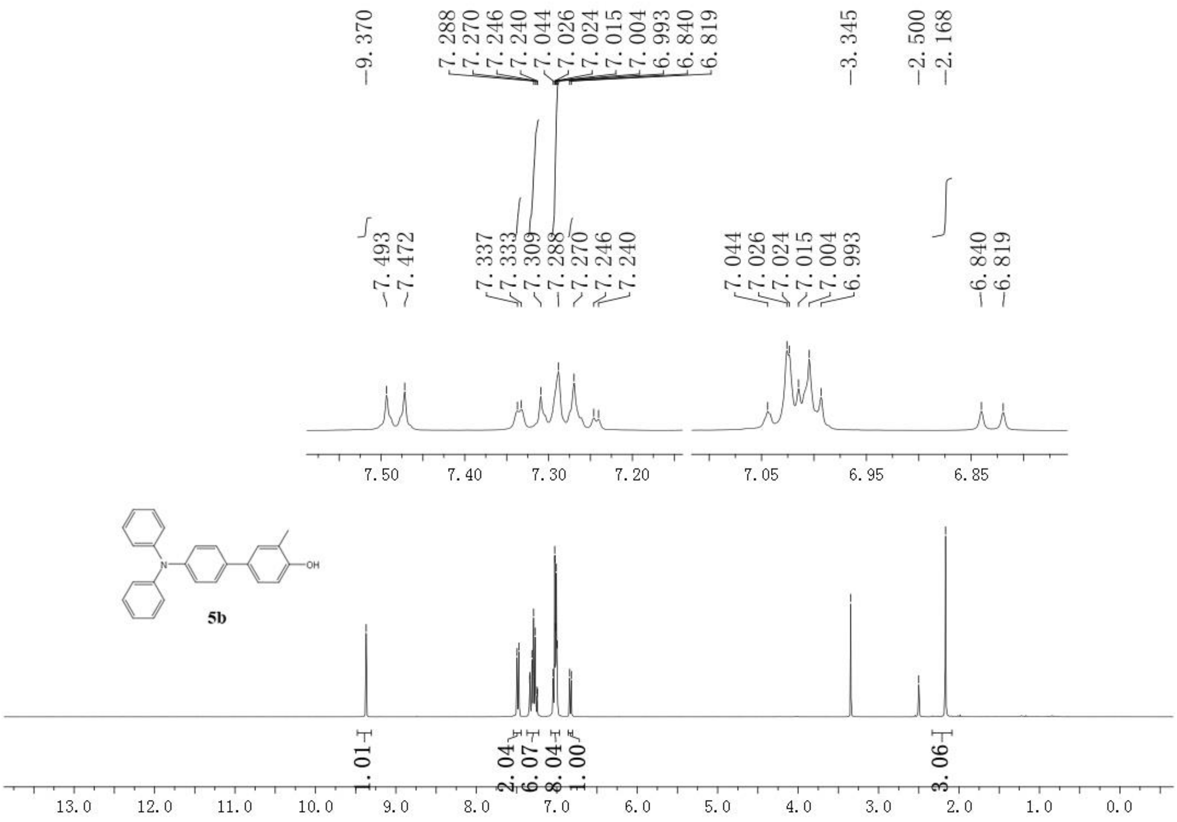

9.370
7.288
7.270
7.246
7.240
7.044
7.026
7.024
7.015
7.004
6.993
6.840
6.819
3.345
2.500
2.168
7.493
7.472
7.337
7.333
7.309
7.288
7.270
7.246
7.240
7.044
7.026
7.024
7.015
7.004
6.993
6.840
6.819
7.50 7.40 7.30 7.20
7.05 6.95 6.85
OH
N
5b
1.01
2.04
6.07
8.04
1.00
3.06
13.0 12.0 11.0 10.0 9.0 8.0 7.0 6.0 5.0 4.0 3.0 2.0 1.0 0.0

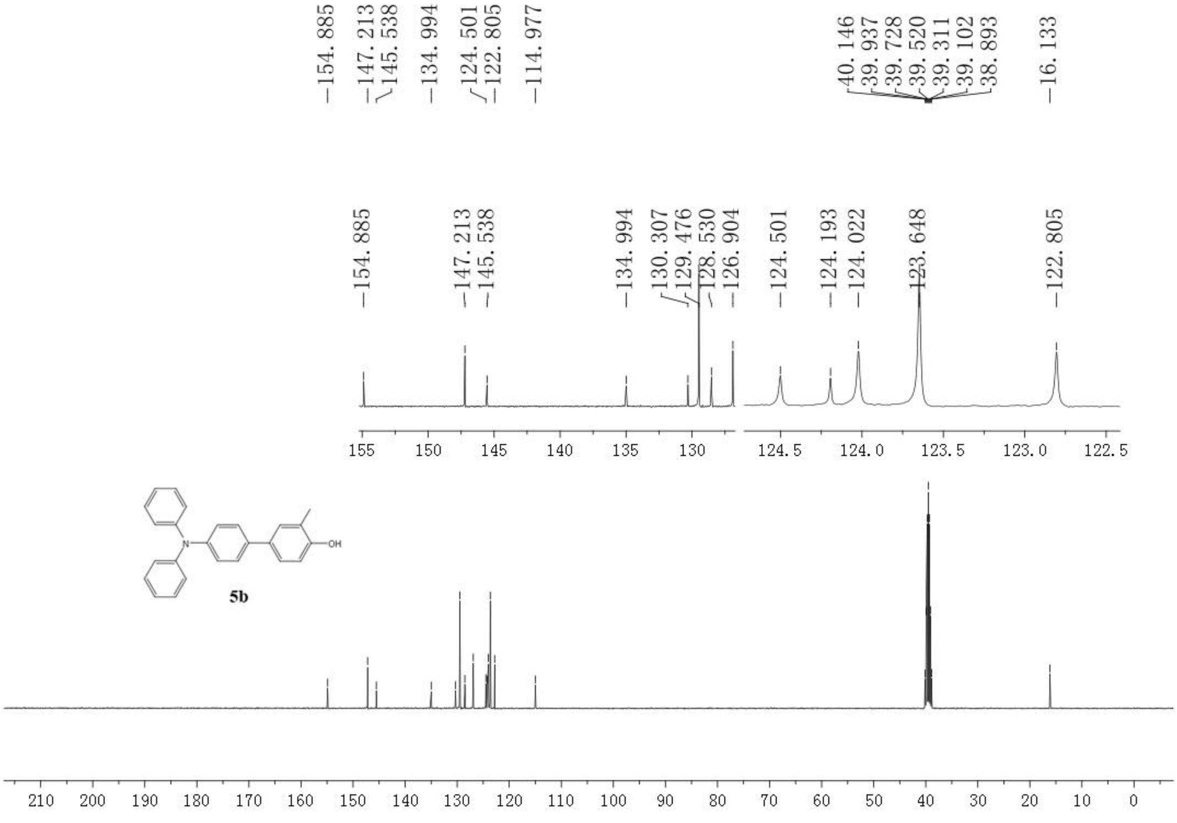

154.885
147.213
145.538
134.994
124.501
122.805
114.977
40.146
39.937
39.728
39.520
39.311
39.102
38.893
16.133
154.885
147.213
145.538
134.994
130.307
129.476
128.530
126.904
124.501
124.193
124.022
123.648
122.805
155 150 145 140 135 130
124.5 124.0 123.5 123.0 122.5
OH
N
5b
210 200 190 180 170 160 150 140 130 120 110 100 90 80 70 60 50 40 30 20 10 0

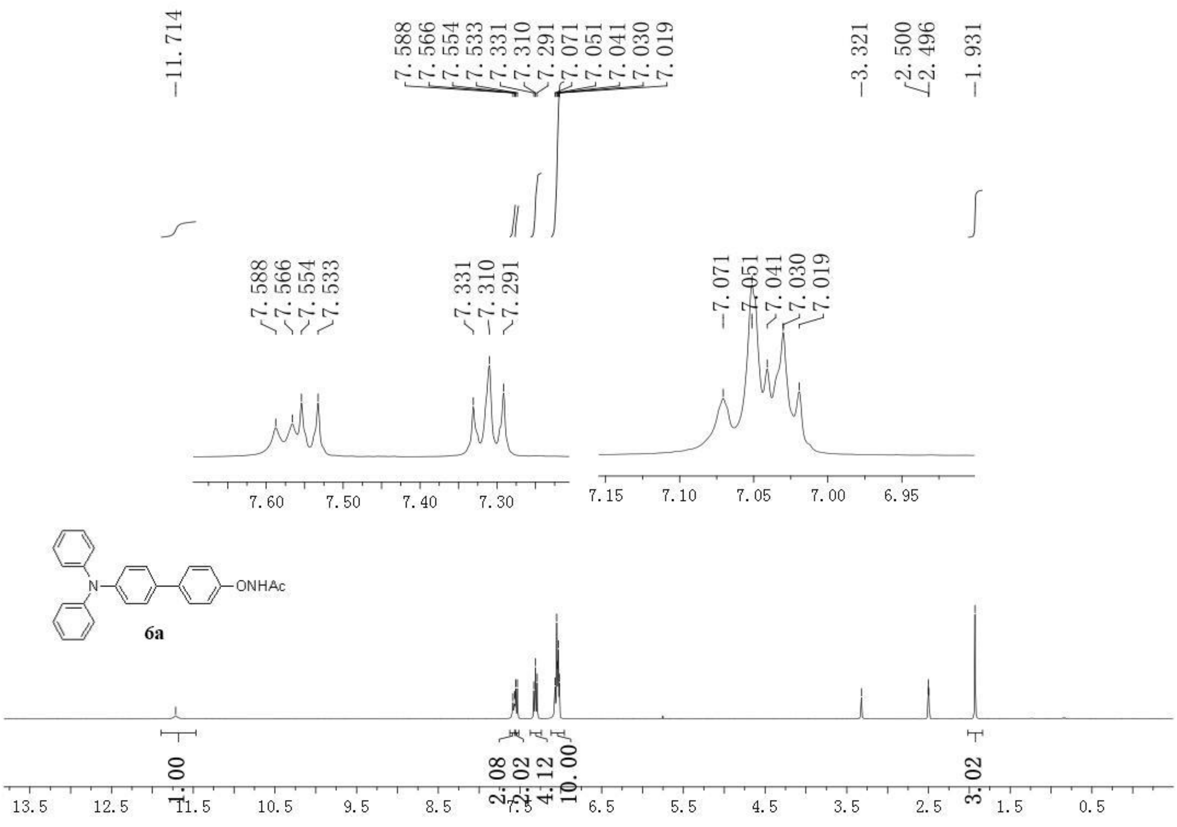

11.714
7.588
7.566
7.554
7.533
7.331
7.310
7.291
7.071
7.051
7.041
7.030
7.019
3.321
2.500
2.496
1.931
ONHAc
6a
1.00
2.08
2.02
4.12
10.00
3.02

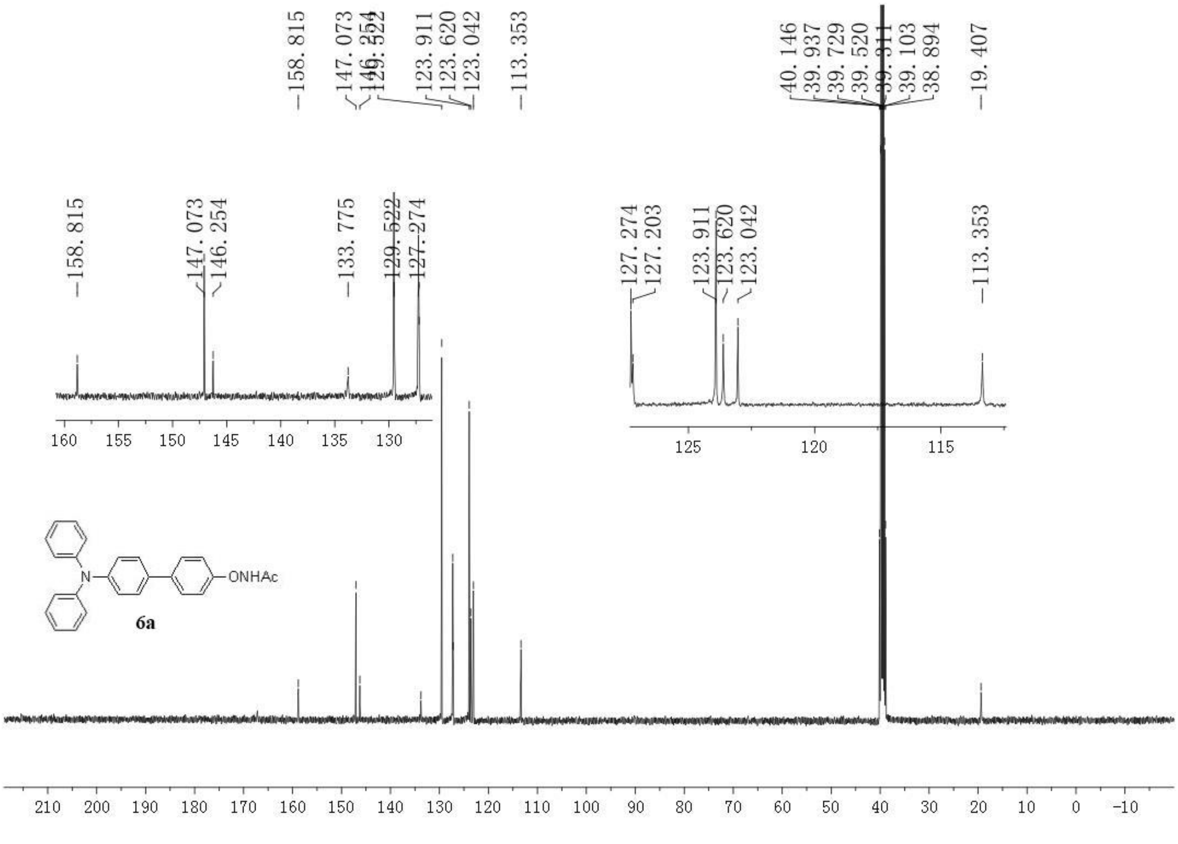

158.815
147.073
146.254
133.775
129.522
127.274
127.203
123.911
123.620
123.042
113.353
40.146
39.937
39.729
39.520
39.311
39.103
38.894
19.407
ONHAc
6a

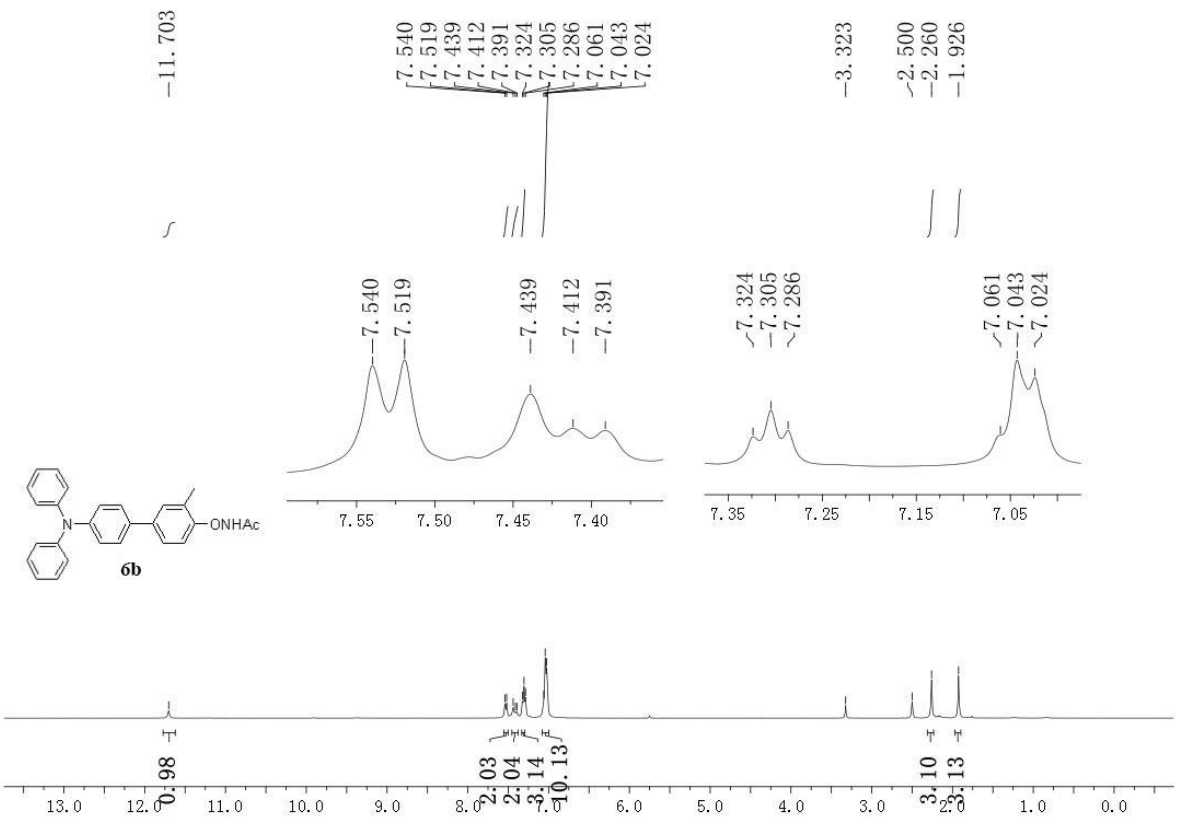
11.703
7.540
7.519
7.439
7.412
7.391
7.324
7.305
7.286
7.061
7.043
7.024
3.323
2.500
2.260
1.926
ONHAc
6b
0.98
2.03
2.04
3.14
10.13
3.10
3.13

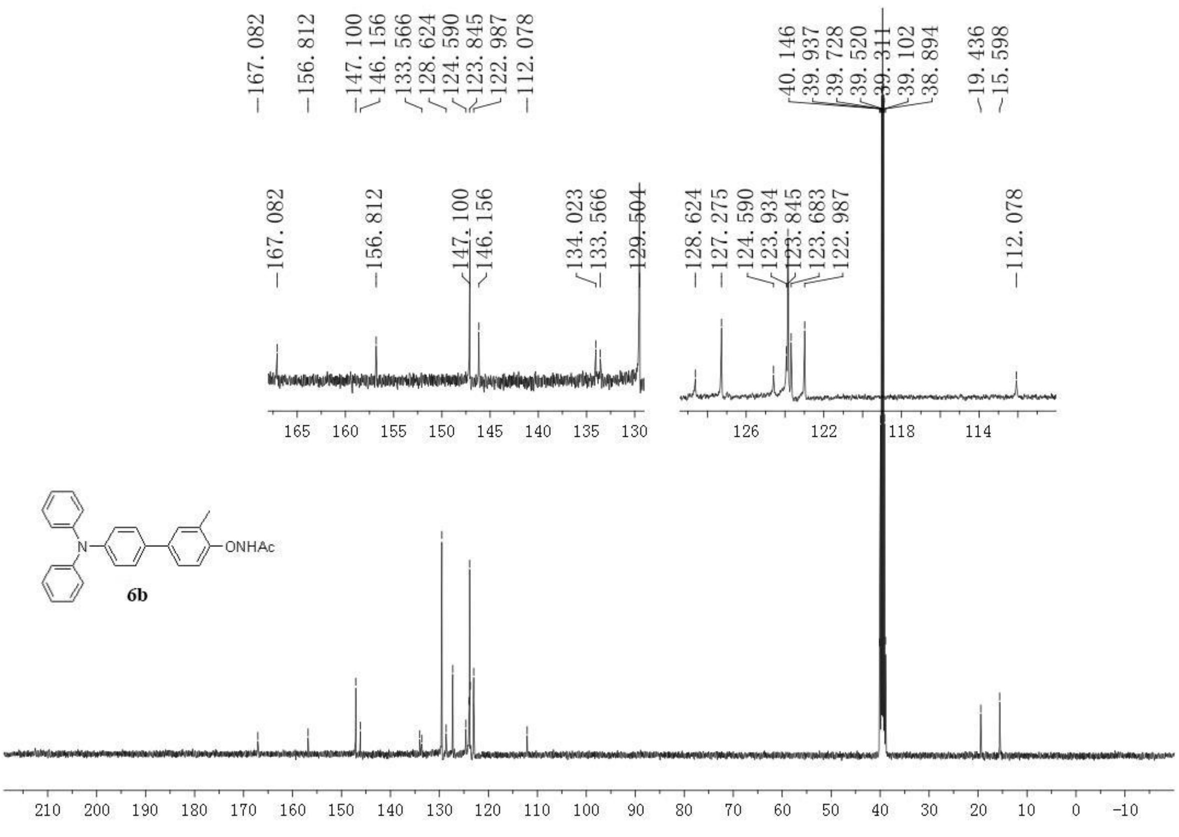
167.082
156.812
147.100
146.156
133.566
128.624
124.590
123.845
122.987
112.078
40.146
39.937
39.728
39.520
39.311
39.102
38.894
19.436
15.598
134.023
129.504
127.275
123.934
123.683
ONHAc
6b

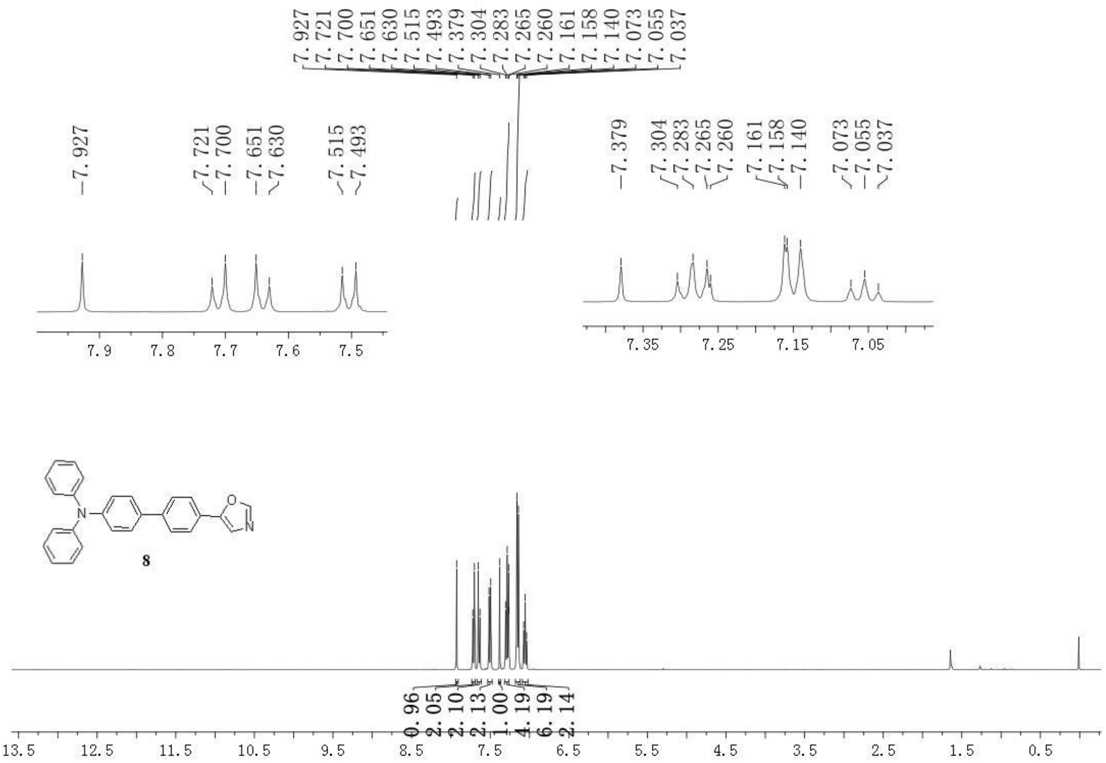
7.927 7.721 7.700 7.651 7.630 7.515 7.493 7.379 7.304 7.283 7.265 7.260 7.161 7.158 7.140 7.073 7.055 7.037
0.96 2.05 2.10 2.13 1.00 4.19 6.19 2.14
8

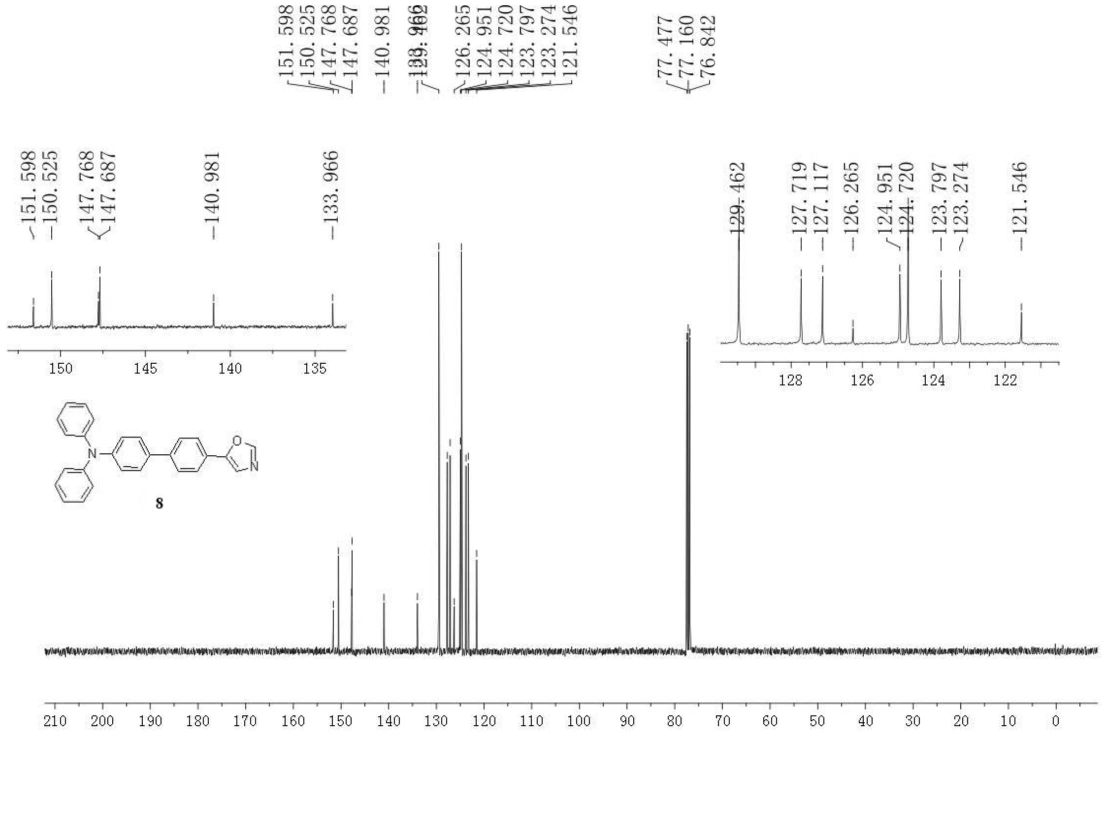
151.598 150.525 147.768 147.687 140.981 133.966 129.462 127.719 127.117 126.265 124.951 124.720 123.797 123.274 121.546
77.477 77.160 76.842
8

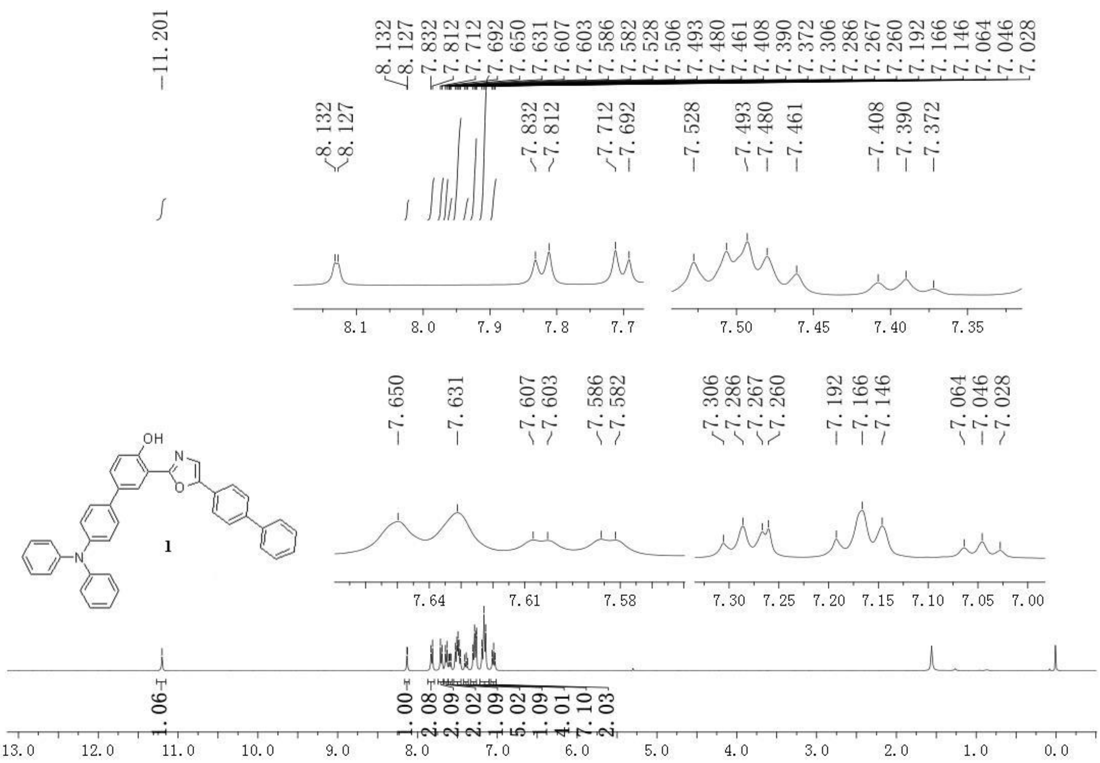
11.201
8.132 8.127 7.832 7.812 7.712 7.692 7.650 7.631 7.607 7.603 7.586 7.582 7.528 7.506 7.493 7.480 7.461 7.408 7.390 7.372 7.306 7.286 7.267 7.260 7.192 7.166 7.146 7.064 7.046 7.028
OH
N
O
N
1
1.06
1.00
2.08
2.09
2.02
1.09
5.02
1.09
4.01
7.10
2.03
13.0 12.0 11.0 10.0 9.0 8.0 7.0 6.0 5.0 4.0 3.0 2.0 1.0 0.0

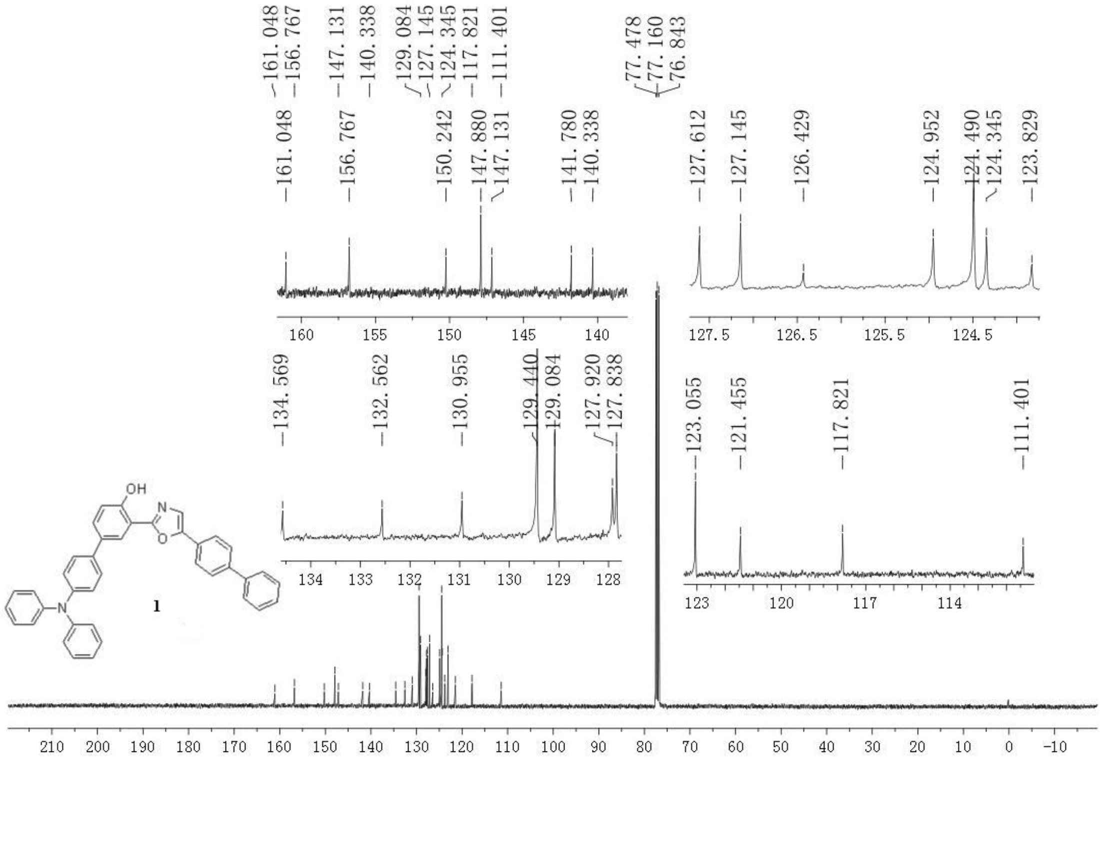
161.048 156.767 147.131 140.338 129.084 127.145 124.345 117.821 111.401
77.478 77.160 76.843
161.048 156.767 150.242 147.880 147.131 141.780 140.338
127.612 127.145 126.429 124.952 124.490 124.345 123.829
134.569 132.562 130.955 129.440 129.084 127.920 127.838
123.055 121.455 117.821 111.401
OH
N
O
N
1
210 200 190 180 170 160 150 140 130 120 110 100 90 80 70 60 50 40 30 20 10 0 -10

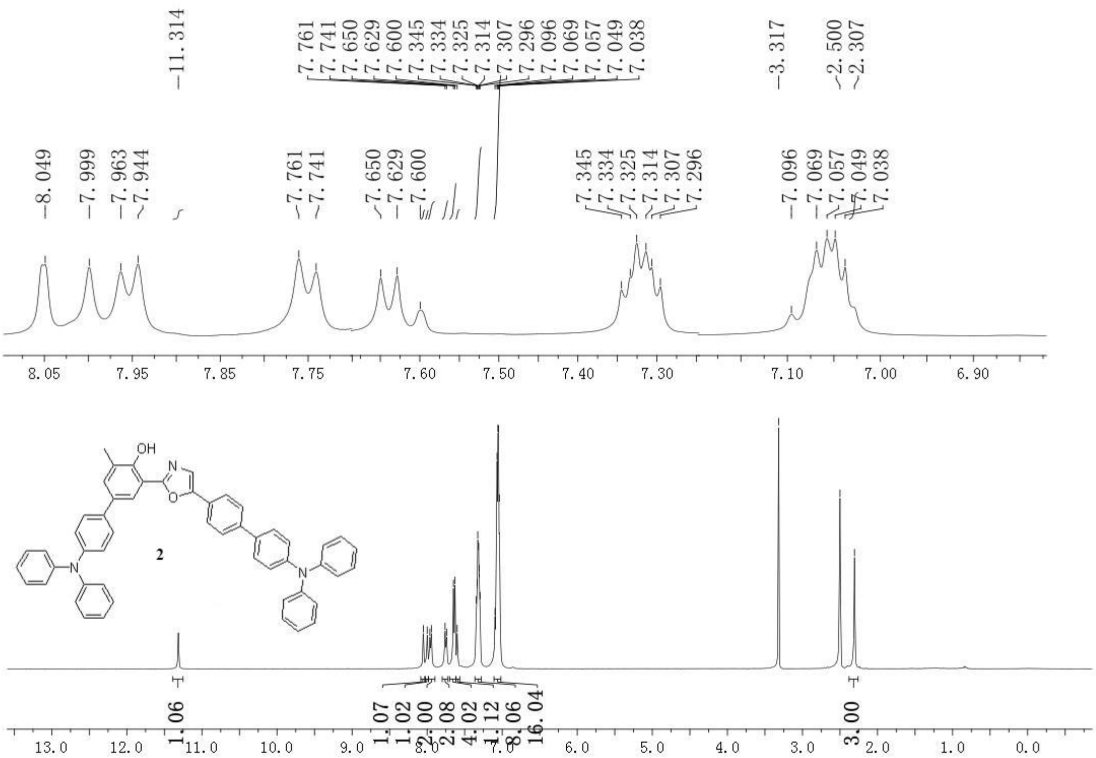
11.314
7.761 7.741 7.650 7.629 7.600 7.345 7.334 7.325 7.314 7.307 7.296 7.096 7.069 7.057 7.049 7.038
3.317
2.500 2.307
8.049 7.999 7.963 7.944
7.761 7.741
7.650 7.629 7.600
7.345 7.334 7.325 7.314 7.307 7.296
7.096 7.069 7.057 7.049 7.038
8.05 7.95 7.85 7.75 7.60 7.50 7.40 7.30 7.10 7.00 6.90
OH
N
O
N
N
2
1.06
1.07 1.02 2.00 2.08 4.02 1.12 8.06 16.04
3.00
13.0 12.0 11.0 10.0 9.0 8.0 7.0 6.0 5.0 4.0 3.0 2.0 1.0 0.0

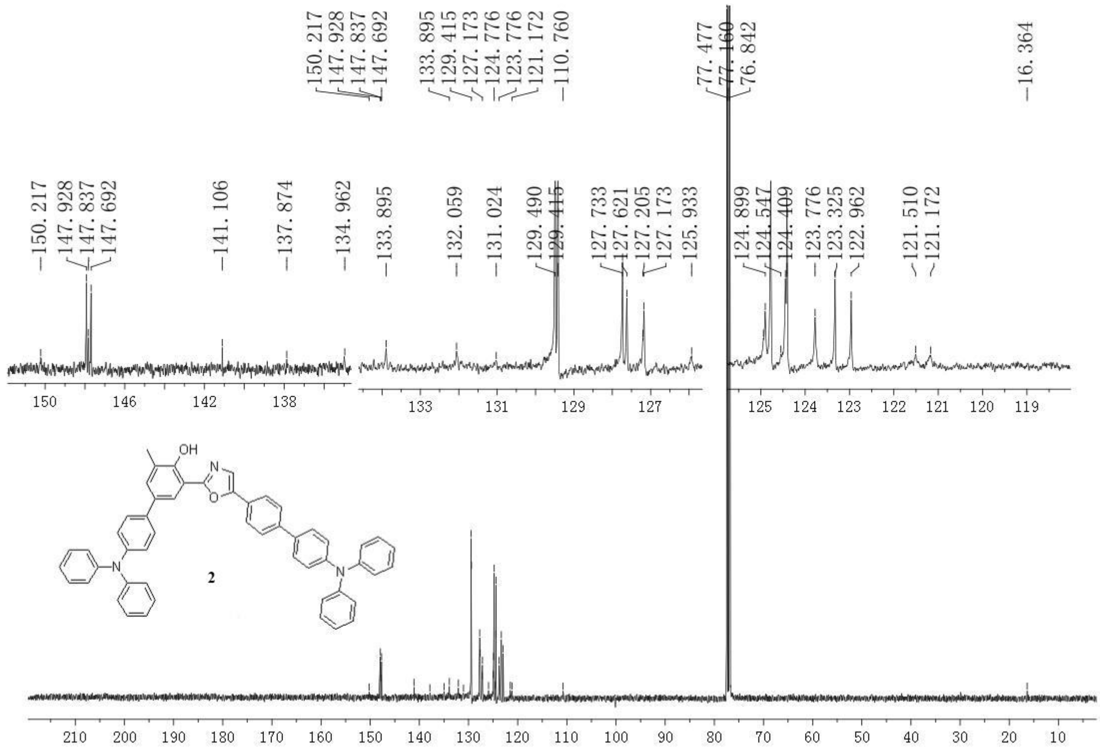
150.217 147.928 147.837 147.692
133.895 129.415 127.173 124.776 123.776 121.172 110.760
77.477 77.160 76.842
16.364
150.217 147.928 147.837 147.692
141.106
137.874
134.962
133.895
132.059
131.024
129.490 129.415
127.733 127.621 127.205 127.173
125.933
124.899 124.547 124.409
123.776 123.325 122.962
121.510 121.172
150 146 142 138
133 131 129 127
125 124 123 122 121 120 119
OH
N
O
N
N
2
210 200 190 180 170 160 150 140 130 120 110 100 90 80 70 60 50 40 30 20 10
